# Coordinated Progression through Two Subtranscriptomes Underlies the Tachyzoite Cycle of *Toxoplasma gondii*

**DOI:** 10.1371/journal.pone.0012354

**Published:** 2010-08-26

**Authors:** Michael S. Behnke, John C. Wootton, Margaret M. Lehmann, Josh B. Radke, Olivier Lucas, Julie Nawas, L. David Sibley, Michael W. White

**Affiliations:** 1 Department of Veterinary Molecular Biology, Montana State University, Bozeman, Montana, United States of America; 2 Departments of Molecular Medicine and Global Health, University of South Florida, Tampa, Florida, United States of America; 3 Computational Biology Branch, National Center for Biotechnology Information, National Library of Medicine, National Institutes of Health, Bethesda, Maryland, United States of America; 4 Department of Molecular Microbiology, Washington University School of Medicine, St. Louis, Missouri, United States of America; INSERM U1016, Institut Cochin, France

## Abstract

**Background:**

Apicomplexan parasites replicate by varied and unusual processes where the typically eukaryotic expansion of cellular components and chromosome cycle are coordinated with the biosynthesis of parasite-specific structures essential for transmission.

**Methodology/Principal Findings:**

Here we describe the global cell cycle transcriptome of the tachyzoite stage of *Toxoplasma gondii*. In dividing tachyzoites, more than a third of the mRNAs exhibit significant cyclical profiles whose timing correlates with biosynthetic events that unfold during daughter parasite formation. These 2,833 mRNAs have a bimodal organization with peak expression occurring in one of two transcriptional waves that are bounded by the transition into S phase and cell cycle exit following cytokinesis. The G1-subtranscriptome is enriched for genes required for basal biosynthetic and metabolic functions, similar to most eukaryotes, while the S/M-subtranscriptome is characterized by the uniquely apicomplexan requirements of parasite maturation, development of specialized organelles, and egress of infectious daughter cells. Two dozen AP2 transcription factors form a series through the tachyzoite cycle with successive sharp peaks of protein expression in the same timeframes as their mRNA patterns, indicating that the mechanisms responsible for the timing of protein delivery might be mediated by AP2 domains with different promoter recognition specificities.

**Conclusion/Significance:**

Underlying each of the major events in apicomplexan cell cycles, and many more subordinate actions, are dynamic changes in parasite gene expression. The mechanisms responsible for cyclical gene expression timing are likely crucial to the efficiency of parasite replication and may provide new avenues for interfering with parasite growth.

## Introduction

Apicomplexan parasites are important pathogens of humans and domestic animals that cause diseases with widespread impacts on global health. The life cycles of these obligate intracellular parasites are complex, involving multiple proliferative and non-growing stages that ensure successful parasite transmission. Pathogenesis, virulence, and disease severity are critically influenced by asexual stage growth rates that can lead to increased parasite biomass and significant tissue destruction and inflammation [1], [2], [3], [4], [5], [6]. Consequently, control of the proliferative stages is vital to combat apicomplexan diseases yet treatments are limited due to the lack of effective vaccines and a paucity of stable drug therapies. In part, the solution to this problem lies in a better understanding of apicomplexan biology, and it is hoped that new post-genomic resources for these parasites will speed the pace of research and open new avenues of therapeutic development.

Apicomplexans have evolved remarkably diverse strategies for intracellular replication of parasites in host tissues. Some species, like the *Toxoplasma gondii* asexual stages studied here, form daughter cells after each round of chromosome replication (endodyogeny). This apicomplexan process differs from the binary fission typical of most eukaryotes in that the daughter parasites form by internal budding within the maternal cell which is consumed by the process. Other apicomplexans may copy their chromosomes and form multiple nuclei, while delaying cytokinesis until the last round of nuclear division or may wait to package haploid genome units into individual nuclei during the final internal budding of new daughter parasites (*e.g.* schizogony in *Plasmodium* or endopolygeny in *Sarcocystis*, respectively) [7], [8].

It is estimated that eukaryotic cells express more than 2,000 enzymes and structural proteins during growth and division with many genes showing a timely progression of transcription through the cell cycle (reviewed in [9]). In the Apicomplexa, it is clear from microarray, SAGE, and proteomic experiments that changes in mRNA and protein levels are extensive during growth and development [10], [11], [12], [13], [14]. Nevertheless, there is uncertainty about the balance of regulatory mechanisms involved and the relative roles of the epigenome [15], [16], the proteome [17], and transcriptional machinery [18], [19], [20]. Earlier concerns that very few specific transcription factors could be recognized in apicomplexan genomes by sequence homology [18], [19], [20] have been mitigated by the discovery of an abundant class of DNA binding proteins related to plant AP2 transcription factors [21].

Here we provide a global view of the cell cycle transcriptome of the *Toxoplasma gondii* tachyzoite replication system during synchronized growth in human fibroblasts. This system has emerged as a major experimental model for the study of apicomplexan cell division [7], [8], [22]. Following invasion and the establishment of the intracellular parasitophorous vacuole, tachyzoites develop through successive coordinated binary divisions within the vacuole to typically form 32 or 64 cells before egress of daughter parasites. The major changes in genomic DNA content, estimates of the length of the S, M and G1 phases, and some details of the morphogenetic progression of nuclear and organelle division and daughter parasite maturation have been determined (reviewed in [7], [8], [22]). We show here that two distinct transcriptional waves involving cyclic expression of at least 2,833 genes accompany parasite replication. These waves functionally separate the expression of conserved eukaryotic genes during the G1 phase from the expression of apicomplexan-specific functions that predominates during the S phase and mitotic periods. This division of transcriptional focus closely mirrors the intimate morphogenetic relationship that has evolved between mitosis and building of the daughter parasites and invasion organelles. Moreover, the importance of promoter mechanisms is indicated by the serial expression of 24 uniquely apicomplexan AP2 transcription factors that exhibit cyclic mRNA dynamics and sharp peaks of protein abundance in close concert with the successive cell cycle processes of tachyzoite growth and division.

## Results

### Synchronization of the *Toxoplasma* tachyzoite cell cycle

**Figure 1:**
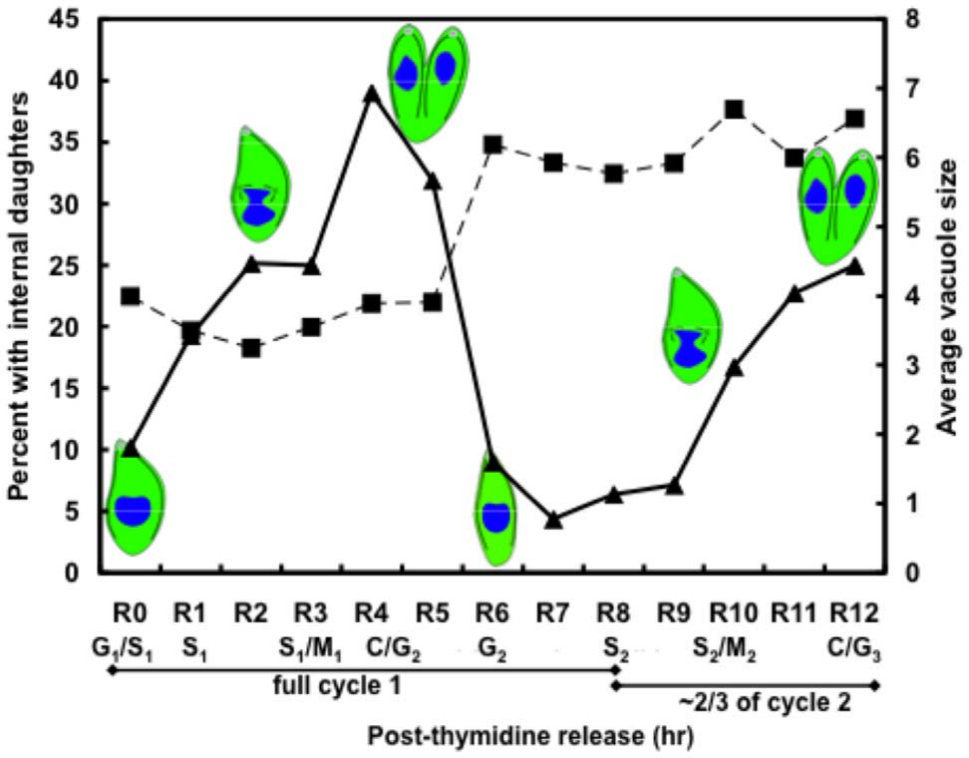
Synchronization of tachyzoite populations by reversible thymidine-inhibition. The average vacuole size (black boxes) and fraction of parasites containing internal daughters (black triangles) were determined in RH^TK+^ parasite cultures growth-inhibited by thymidine. At hour intervals through 12 h post-thymidine release, infected monolayers were fixed and stained with anti-IMC1. Vacuole size and the internal daughter fraction were determined in 100 vacuoles selected at random. The synchronous division of these populations is evident by the cyclical rise and fall of internal daughter forms that is accompanied by a stepwise increase in vacuole size that marks the end of one cycle and the start of the next. The cell cycle progression shown here was estimated from these experiments and is validated by previous studies using this synchrony model [23], [25].

To characterize the cell cycle transcriptome of *T. gondii* tachyzoites, we expanded a thymidine-synchrony model [23] to isolate sufficient RNA for microarray expression studies. Tachyzoites from 10–20 infected flasks were harvested each hour post-thymidine release for RNA isolation. The time course samples were collected out of series and in duplicate to minimize experimental variance. A small portion of each pooled population was fixed for genomic DNA analysis by flow cytometry (Fig. S1), and in parallel cultures we assessed internal daughter formation and the timing of division by immunofluorescence assay. Thymidine treatment of RH^TK+^ tachyzoites [23] results in tight synchrony that is remarkably stable in replicate cultures. Thus, the expected synchronous cell cycle progression was achieved in pooled populations as first described for single cultures [23]. *Toxoplasma* tachyzoite populations blocked by thymidine and then released satisfy stringent criteria for validating growth synchrony (as defined in Appendix 1 of [24]). Genomic content of the released populations followed a time-ordered progression permitting nearly two full synchronous divisions before encountering significant host cell lysis (12 h total timeframe, Figs. 1 and S1). Internal daughter formation was cyclical with budding fractions rising to nearly half the population following thymidine-release before falling to <5% and then rising again as parasites progressed through the second S phase (Fig. 1, internal daughters). Average vacuole size increased stepwise in thymidine-synchronized populations immediately following the maximum time of internal daughter formation (Fig. 1) and paralleled the rise in haploid parasites (Fig. S1). Finally, the increase in parasite numbers was accompanied by predictable changes in cell size based on forward scatter analysis by flow cytometry analysis with large parasites present in S phase and mitosis (post-release 2–4 h) becoming smaller as daughters resolve their mother at the end of cytokinesis (post-release 5–6 h) (not shown, see [25]).

### Whole-cell expression profiles reveal two transcription waves accompany each parasite division

The ToxoGeneChip microarray (http://ancillary.toxodb.org/docs/Array-Tutorial.html) comprised of ∼8,000 *Toxoplasma*-specific probe sets (eleven 25 mer probes/gene) was used to measure mRNA expression in 13 duplicate samples (R0 to R12, Fig. 1) covering 12 hours post-synchronization and nearly two tachyzoite replication cycles. Genes showing a cyclical expression pattern were identified rigorously by two methods: first a criterion of significantly elevated variance was applied to samples R0 to R7 (one full cycle) that identified 3,518 candidates (ANOVA FDR = 0.1); second a cubic B-spline criterion with empirical confidence intervals determined from 1000 simulations (see Methodssection and Fig. S2; also [26], [27], [28]) was applied to the entire 12 hour time course and conservatively reduced these candidates to 2,869 probe sets (2,833 uniquely defined genes) that have significant peaks or troughs of mRNA abundance separated by an interval of 7.4–8.7 hours. These conservative criteria, together with the non-serial strategy of parasite sample collection, serve to minimize artifacts arising from systematic errors, autocorrelation, or uncertainties about the background data model. Such artifacts have been held responsible for overestimates of the number of cycling genes in synchronized yeast models [29], [30]. To these 2,833 genes, ∼250 additional genes had a significant peak and/or trough of mRNA abundance during the first cell cycle but did not repeat cyclically in the second, possibly reflecting developmental mechanisms that accompany tachyzoite growth. Thus, 40% of all coding sequences annotated in the *Toxoplasma* genome exhibited significant cell cycle expression patterns.

**Figure 2:**
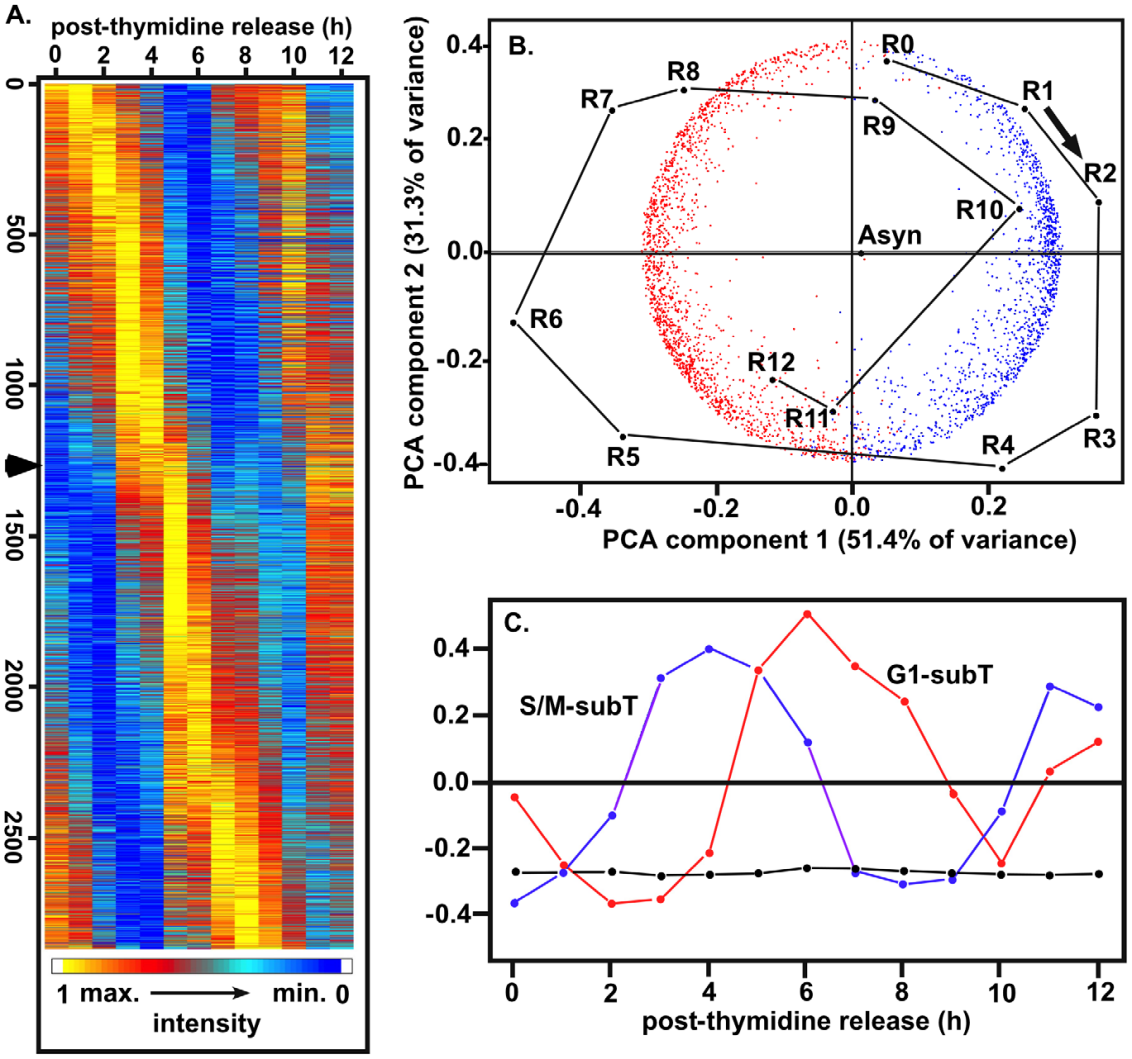
Global transcript analysis of the tachyzoite division cycle. Duplicate RNA samples from each hour post-thymidine (0–12 h plus asynchronous samples) were used to synthesize cRNA probes for hybridization to the *Toxoplasma* Affymetrix GeneChip. Probe sets showing significantly increased variance through the cell cycle were identified by ANOVA analysis (10% false discovery) and further resolved by cubic B-spline modeling. (**A**) Heat map display of the 2,833 genes judged to be significantly cyclical was ordered by the times of the first principal maximum of RNA abundance in the first cell cycle, as estimated from the continuous cubic B-spline models of the time series for each gene. (**B**) Principal components analysis (PCA) provides objective evidence, following the logic described in the Results section, for the predominant cyclic expression series of the 2,833 genes (red and blue points) and the 13 time points (black), and for 2 predominant subtrancriptomes. Plots are superimposed of the 2-dimensional projections from PCA of the 2,833×2,833 between genes covariance matrix and the 14×14 between samples covariance matrix (the 13 time points R0 to R12 plus the asynchronous control labeled ‘Asyn’). The time axis circle runs clockwise, starting at the G1/S transition (R0) at the top, with the first S phase (R0-R2) corresponding to S phase in the second cycle (R8-R10) and the mitosis/cytokinesis of R4-R5 recurring at R11-R12. The cycle of synchronized sample points, especially in the partial second cycle, tends to converge slightly towards the ‘Asyn’ center as the time course proceeds, consistent with slight progressive loss of synchrony during the experiment. The red and blue gene points correspond respectively to the G1 and S/M subtranscriptomes, partitioned at 4.6 hours according to the criteria described in the Results section. (**C**) Singular value decomposition (SVD) analysis objectively reveals a cyclic process corresponding to the temporal G1 and S/M subtranscriptomes. Following the logic described in the Results and Methodssections, this analysis provides quantitative estimates of the RNA proportions, aggregated over genes, attributable to the non-cyclic steady-state expression level present throughout the cell cycle (component 1, black) and to the cyclically varying components of the two subtranscriptomes (components 2 and 3, red and blue). Smaller components of SVD, not shown, also identify small subsets of genes that have atypical profiles, but this analysis fails to find further major subclusters within the subtranscriptomes.

The mathematical structure of the data provides an unbiased verification of cyclic mRNA expression patterns along the 13 time points. Fig. 2A is a heat map of the 2,833 genes ordered by the times of their peak mRNA abundances in the first cell cycle, as estimated from the continuous cubic B-spline models of each gene's time series. As can be seen from this map, strong peaks of mRNA abundance recur in the second cell cycle after an approximate 8 hour interval, although a minority of the genes show reduced second cycle amplitudes and/or deviations from exact periodicity which may reflect developmental processes and some expected de-synchronization during the progression of this population. The predominant cyclic pattern is confirmed by principal components analysis (PCA, Fig. 2B), which co-plots the 2-dimensional covariance projections of the 2,833 genes (red and blue points) and the 13 time points (black points; R0-12, asyn = asynchronous populations). The order and cyclic series of the time points emerge objectively from this PCA alone (Fig. 2B points R0-R12), without considering *a priori* information from the experimental design. Given this experimental information, it is clear that the time axis circle runs clockwise, starting at the G1/S transition (R0) at the top. The first S phase (R0-R2) corresponds to S phase in the second cycle (R8-R10) and the mitosis/cytokinesis of R4-R5 recurs at R11-R12. The data from asynchronous samples (labeled ‘Asyn’) compute onto the center of the circle and the synchronized sample points, especially in the partial second cycle, tend to converge slightly towards this center as the time course proceeds, consistent with some progressive loss of synchrony during the experiment.

**Figure 3:**
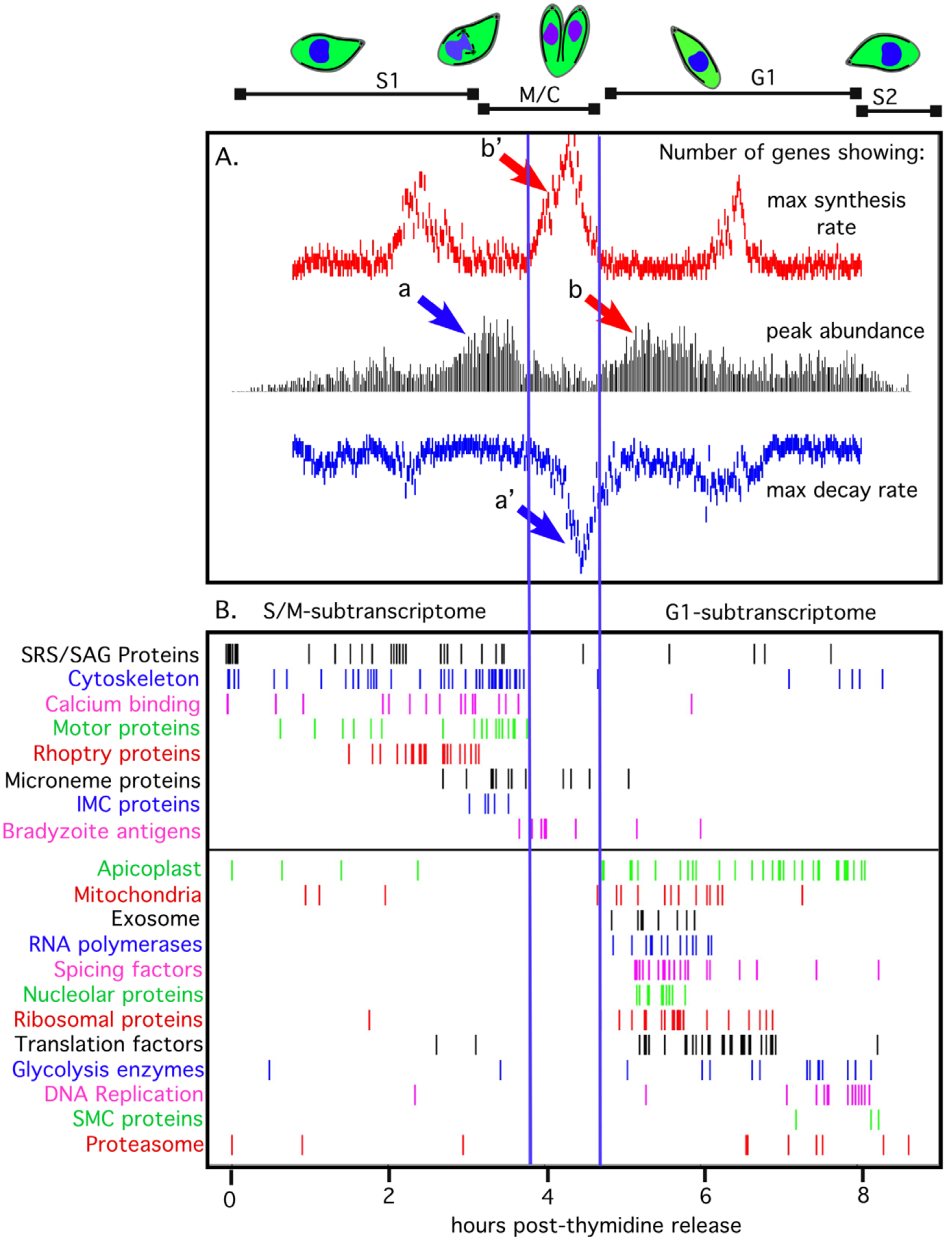
Genes encoding basal or specialized apicomplexan functions have different transcriptional dynamics in the tachyzoite cell cycle and define two functional subtranscriptomes. (**A**) Two major waves were apparent in the peak times of RNA abundance, corresponding to the S/M and G1 subtranscriptomes and separated by a transition phase of active RNA synthesis and decay. Histograms represent the numbers of genes (counted in 2-minute bins) showing maximum net increase in mRNA change (red), maximum RNA abundance (black), and maximum net rate of RNA decrease (blue, inverted scale) through the first complete parasite division cycle (0–8.7 h post-thymidine release). The maxima were computed from derivatives of the spline model curves (methods section). The peaks of RNA abundance (black histogram) showed the two major broad waves corresponding to the two subtranscriptomes. The genes in the black histogram S/M peak labeled a mostly showed maximum net decrease in mRNA levels in the later transition phase blue peak labeled a′. The genes in the black histogram G1 peak labeled b mostly derived from the prior transition phase red peak of maximum net increase in mRNA levels labeled b′. Thus, the transition phase is characterized by rapid increases and decreases in the amounts of many different transcripts, coincident with the internal budding and G1 initiation of daughter cells and the consumption of the mother cell. (**B**) The peak times of mRNAs in specific functional groups are partitioned serially into the two subtranscriptomes. The G1 Subtranscriptome comprised 1,146 probe sets, 54% of which can be annotated functionally by homology using current databases. The S/M-subtranscriptome contained 1,493 probe sets, of which 40% can be annotated functionally. Thus, the S/M subtranscriptome encodes a substantially greater proportion of ‘hypothetical proteins’ with no current functional homologs, which are either unique to *Toxoplasma* or conserved in other Apicomplexa. For 228 probe sets, the peak RNA abundance occurred within the 3.8–4.7 hour transition zone with proportions of functionally annotated versus hypothetical proteins resembling the S/M subtranscriptome.

The 2,833 cyclically expressed genes fall into two predominant subtranscriptomes, shown objectively by PCA (Fig. 2B), in which the first two eigenvectors accounted for 83% of the gene/gene covariance, and by singular value decomposition (SVD, Fig. 2C). By inspection of Fig. 2A and Fig. 3A, two classes of genes appeared to lie on each side of a point at ∼4.6 hours after thymidine release (late cytokinesis/early G1) showing as a ‘kink’ in the expression cascade (see arrow in Fig. 2A) and as an intermodal trough in the number genes showing peak mRNA abundance (Fig. 3A, black histogram). When this 4.6 h point was used to divide the 2,833 genes into ‘blue’ and ‘red’ classes, the gene sets partitioned almost exactly at the zero value of the first PCA eigenvector (Fig. 2B, horizontal axis). Thus, PCA provides a mathematical rationale justifying the two predominant subtranscriptomes (G1, red; S/M blue) with a boundary at 4.6 hours. These two subsets of genes also emerged objectively from SVD, represented by the cyclical patterns of the second and third components colored red and blue in Fig 2C, and can also be visualized as the distinct broad modes in the numbers of genes with peak mRNA abundances in G1 and S/M phases (Fig. 3A, black histogram).

From inspection of the cell cycle time courses for individual genes, it appeared that many genes maintained moderate steady-state mRNA levels throughout the cycle, reflecting shallower cyclic mRNA patterns, while relatively fewer genes (*e.g.* rhoptry genes discussed below) had profiles with large amplitudes that declined to the very low levels of expression. SVD analysis can be used to estimate the global proportions of the total mRNA present in these non-cyclic steady state and wave-like cyclic components (using all-gene normalized data, see Methodssection). The largest SVD vector (black line and points, Fig. 2C), representing the non-cyclic steady state fraction aggregated over all 2,833 genes, contained 77.2% of the total mRNA signal. This non-cyclic fraction increased only slightly to 80.8% for the entire set of 7,784 genes, reflecting the generally low or insignificant expression levels found for most of the 4,951 *Toxoplasma* genes that were excluded from the cyclic set by our stringent criteria. The cyclically varying fractions (i.e. the amplitude of the waves superimposed on the steady state, aggregated for all genes) were 7.8% and 5.4% of the total mRNA for the G1 and S/M subtranscriptomes respectively (Fig 2C, red and blue vectors). Intriguingly, very similar global non-cyclic and cyclic mRNA proportions have been found during the cell cycle of budding yeast. Omberg et al (2009)[31] reported 72.3% of the total mRNA non-cyclic, and 8.7% and 6.7% attributable respectively to the main M/G1 and G1/S peaking cyclic components in a synchronized *Saccharomyces cerevisiae* model. Thus, *Toxoplasma* tachyzoites and yeast appear grossly similar in the global transcriptional dynamics of their cell cycles while having many differences in the morphogenetic processes and the crucial molecular components involved.

### The parasite cell cycle divides the renewal of basic biochemical functions from the specialized process of building daughters

The first 8 hours following synchronization by thymidine release represent one complete cycle with >98% of the 2,833 cyclic genes showing a clear peak during this period. Ordering mRNAs by this peak expression time and counting the number of genes with peaks in each time bin (Fig. 3A, black histogram) reveals the two major waves of transcription associated with the tachyzoite S phase through cytokinetic periods (S/M-subtranscriptome) or the major G1 period (G1-subtranscriptome). An active transition phase (3.8–4.7 hours) separates these two waves, characterized by a large wave of rapid net decline in mRNA levels for many other genes with earlier peak expression in S/M phases (Fig. 3A, blue histogram, and waves labeled a′ and a) and a large wave of rapid net synthesis for many genes with mRNA peaks later in G1 (see Fig.3A, red histogram, and peaks labeled b′ and b).

**Figure 4:**
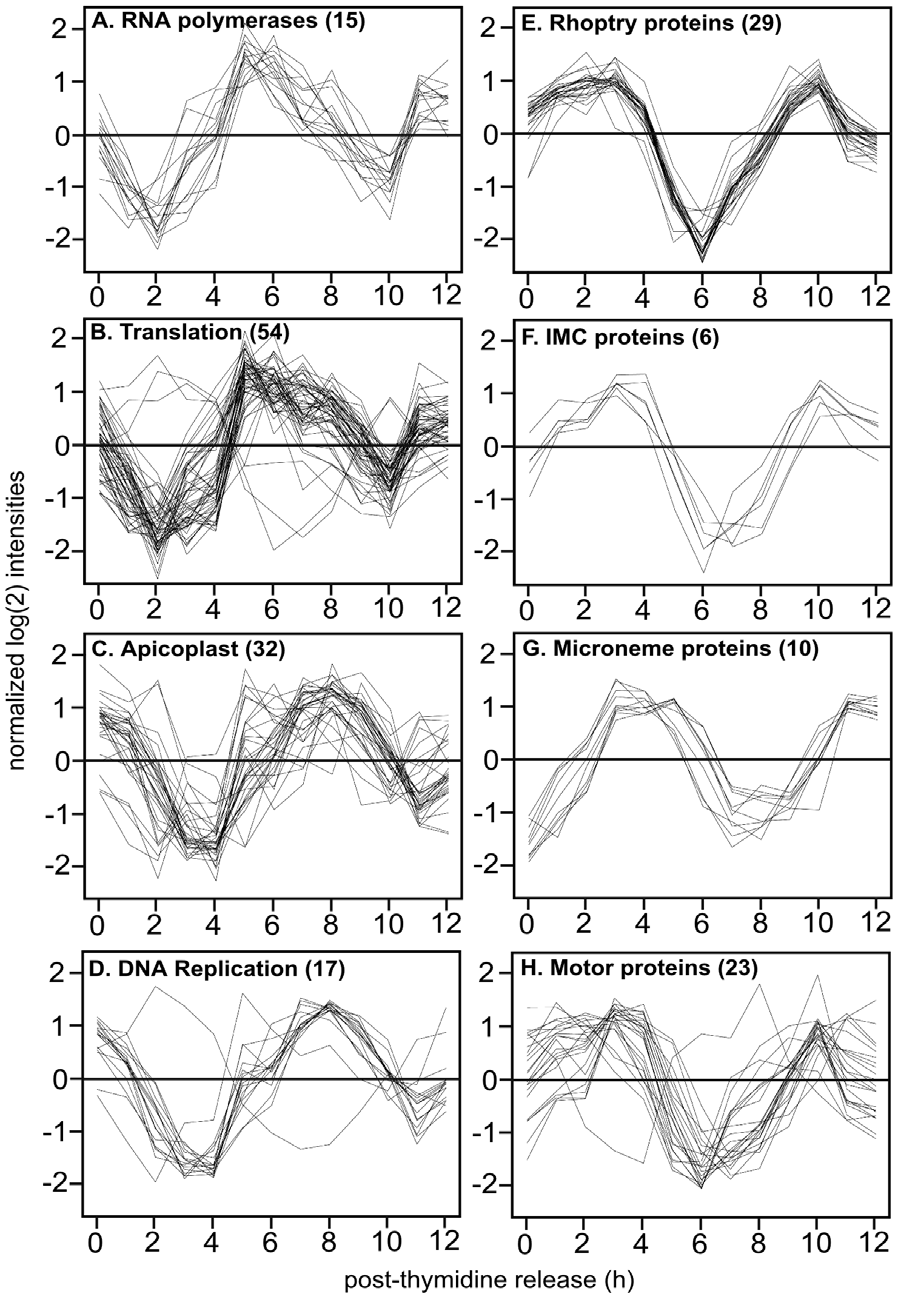
Proteins with similar function define tight clusters of transcript expression patterns within each subtranscriptome. (**A–D**) Progression through the early G1 period is associated with maximum expression of transcripts encoding protein components of transcription and translational processes (5–6 h post-thymidine release) followed in mid-to-late G1 by a tightly clustering of genes involved in many aspects of DNA replication (7–8 h post-release). Apicoplastid genes (nuclear encoded) have a similar late-G1 profile consistent with the timing of plastid division in the next phase of the tachyzoite cell cycle (S phase). (**E–H**) Rhoptry and microneme proteins define distinct tight clusters with sequential timing during late S phase through mitosis. Rhoptry and IMC mRNA expression (35 total genes) peaked in late S phase/early mitosis (2–3 h post-thymidine release and then again at 10 h), which was followed by a rapid and dramatic decline of these transcripts in early G1. Transcripts encoding micronemes (10 genes) along with a number of motor proteins (kinesins and myosins; 23 genes) reach a maximum 1–2 h later as parasites undergo mitosis and complete budding (3–4 h and again at 11 h). The order of gene expression with plastid biogenesis coming before new rhoptry or microneme synthesis correlates with the timing of plastid and organelle replication during tachyzoite proliferation [35].

The genes present in the two main expression waves describe a unique switch in biological focus from genes involved in the renewal of basal biosynthetic functions and metabolism (G1) to specialized apicomplexan processes of daughter maturation and egress (S/M). Specific genes in the first half of the G1-subtranscriptome include mRNAs encoding components of the transcription and translation machinery and basic metabolism and catabolism, while genes required for DNA replication were maximally expressed in the second half of G1 (Fig. 3B). Genes with maximum mRNA levels in the early G1-subtranscriptome included mRNAs encoding 15 RNA polymerases, 16 splicing proteins and 52 factors from the ribosome and translation regulatory complexes (Figs. 3B; 4A and B). Eight components of the cellular exosome responsible for mRNA degradation were also tightly co-expressed in early G1 (Fig. 3B), and increased activity of this complex in this phase could influence the rapid degradation dynamics of mRNAs encoding specialized apicomplexan structures (see discussions below). Genes involved in energy metabolism were also found here with mitochondrial enzymes reaching maximum expression in the first half of G1, while most of the enzymes of the glycolytic pathway had peak timing ordered throughout this cell cycle period (Fig. 3B). DNA replication genes formed a distinct co-regulated cluster that shared nearly identical peak times in late-G1 in advance of commitment to chromosome replication in S phase (Figs. 3B; 4D). Members of this group included primase and extension DNA polymerases, several associated repair enzymes, DHFR-TS as well as the DNA clamp proteins licensing factor C (clamp loader) and proliferating-cell-nuclear-antigens 1 and 2 [32], [33]. Interestingly, some nuclear encoded plastid genes (32 total) were maximum in early G1, while many others peaked in late G1 (Fig. 3B and 4C). The overall timing of plastid gene transcription in the G1 period is consistent with the duplication of this organelle in the next phase (S) of the cell cycle [34].

There is an intimate relationship between mitosis and internal daughter budding in *Toxoplasma* (and other members of Apicomplexa) as new apical complexes of each daughter form in close proximity to the nucleus and the duplicated centrosomes (reviewed in [7], [8], [22]). This intricate biosynthesis of daughter structures is reflected in the order of mRNAs that peak in the S/M-subtranscriptome (Fig. 3B). Levels for mRNAs encoding some surface antigens (SRS and SAG) along with proteins decorating the daughter cell inner-membrane-complex (IMC), cytoskeletal and motor proteins, as well as proteins secreted from rhoptry and microneme invasion organelles reach peak levels during the second half of the tachyzoite cycle (Figs. 3 & 4). For example, mRNAs encoding 29 rhoptry proteins form a distinct cluster (Fig. 4E) with mRNA levels rising to a maximum 2–3 h post-thymidine release (S to M phase) and then dramatically decreasing in early G1 (minimum 6 h) before peaking again in the next S phase (peak 10 h). The patterns of rhoptry mRNAs (encoding ROP secreted protein kinases and neck proteins) were offset by 1–2 h from microneme mRNAs whose co-transcription defined a distinct temporal class (Fig. 4G 1st peak 3–4 h, 2nd peak 10–11 h). Other mRNAs, of both known and unknown functions, shared the cyclic dynamics of rhoptry and microneme classes, suggesting that common transcription and mechanisms that lead to decreases in mRNA abundance may operate (e.g. Fig. 4F IMC proteins and 4H, motor proteins). By contrast dense granule mRNAs largely were not regulated in the tachyzoite cell cycle (12 of 16 GRA mRNAs examined were non-cyclical). The relative timing of organelle transcription with plastid genes transcribed first followed by rhoptries and then micronemes reflects the order in which these organelles are replicated during tachyzoite division [35]. The RH derived strain used in these studies poorly differentiates into bradyzoites and tissue cysts [36], nonetheless, we discovered a few bradyzoite-specific mRNAs were cyclically expressed (TGME49_032940, TGME49_120180, TGME49_120190, TGME49_080570, TGME49_093790, TGME49_002020, TGME49_008730, TGME49_112600) with peak transcription in the late mitotic period (Fig. 3B).

**Figure 5:**
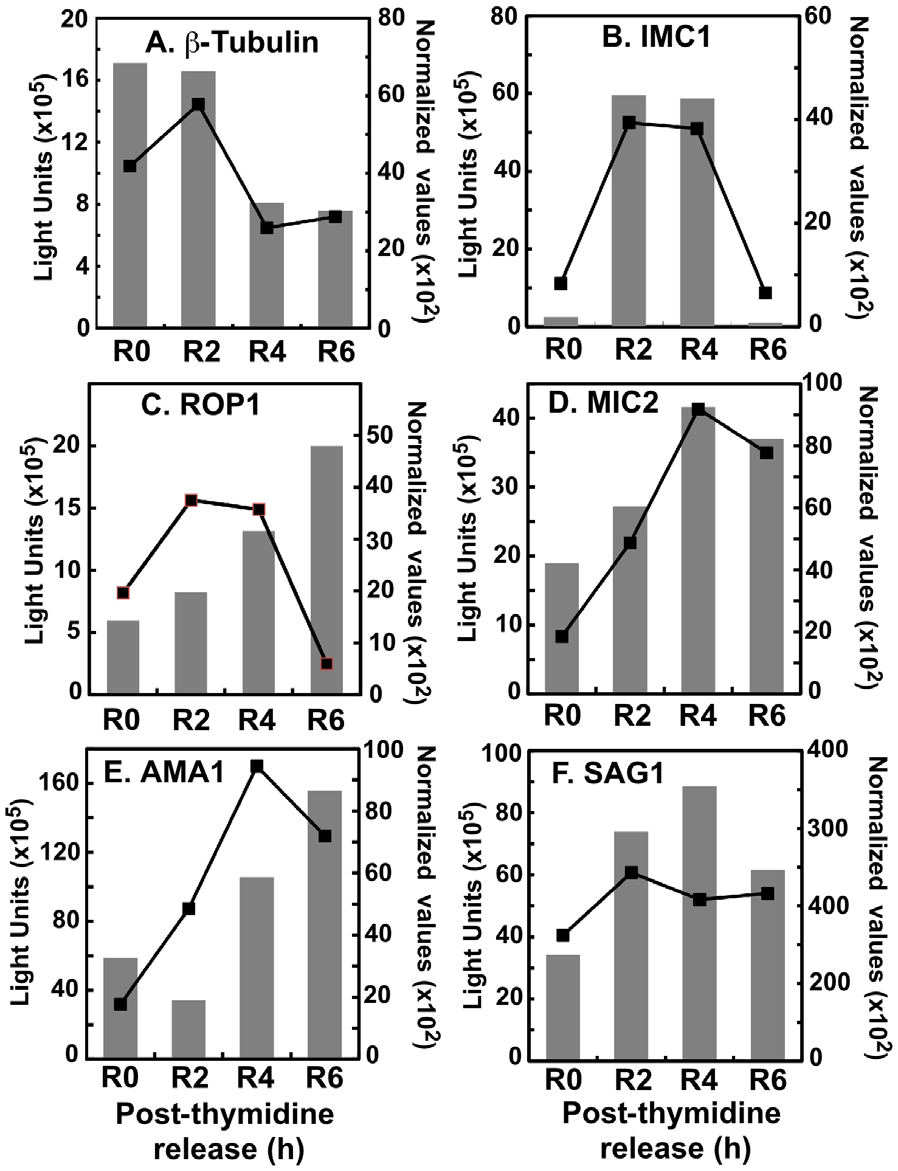
Cyclical changes in transcripts are reflected in the cell cycle regulation of protein levels. Protein accumulation roughly mirrors expression levels during the cell cycle. High protein levels are stably retained in R6 for microneme (MIC2, and AMA1) and rhoptry (ROP1) proteins despite down regulation of transcripts. Synchronized parasites were released and protein lysates generated at the times indicated. Levels of *Toxoplasma* proteins were detected by quantitative western blotting using specific antisera (see methods) and are shown as the average of three replicates (bar graphs). Normalized mRNA expression levels are shown from the microarray data (line plot).

Matching transcriptional changes with respect to protein is challenging owning to the complexity of mechanisms involved, and we have not made a global comparison of mRNA and protein levels. Nevertheless, we examined several proteins encoded by the S/M-subtranscriptome in order to determine whether cyclical mRNA patterns may inform the cell cycle variation of the encoded protein. A comparison of the levels of several proteins quantified from Western blots (grey bars) versus their relative mRNA levels (normalized microarray data, line graphs) in synchronized tachyzoites is presented in Figure 5. Expression of IMC1 which increases during daughter budding, is commonly used to visualize mitotic parasites in *Toxoplasma* cell cycle studies [37]. The sharp changes in IMC1 protein during this period were closely correlated with the cell cycle transcriptome (Fig. 5B), as both mRNA and protein levels for IMC1 were dramatically induced during late S phase (R2), and they rapidly declined in parallel as parasites progressed to complete mitosis and cytokinesis (R6). Coincident cyclic patterns were also observed for two microneme proteins (MIC2 and AMA1) whose levels changed similar to their mRNAs (Figs. 5D and E) as did protein and transcript levels for surface antigen, β-tubulin and SAG1 (Figs. 5A and F). Intriguingly, the increase in rhoptry protein 1 (ROP1) followed the cell cycle induction of ROP1 mRNA, however, ROP1 protein levels did not parallel the rapid decrease of the encoded mRNA (Fig. 5C) consistent with an organelle environment that stores its protein contents for later invasion.

### Cyclical gene expression is linked to specific cell cycle transitions

**Figure 6:**
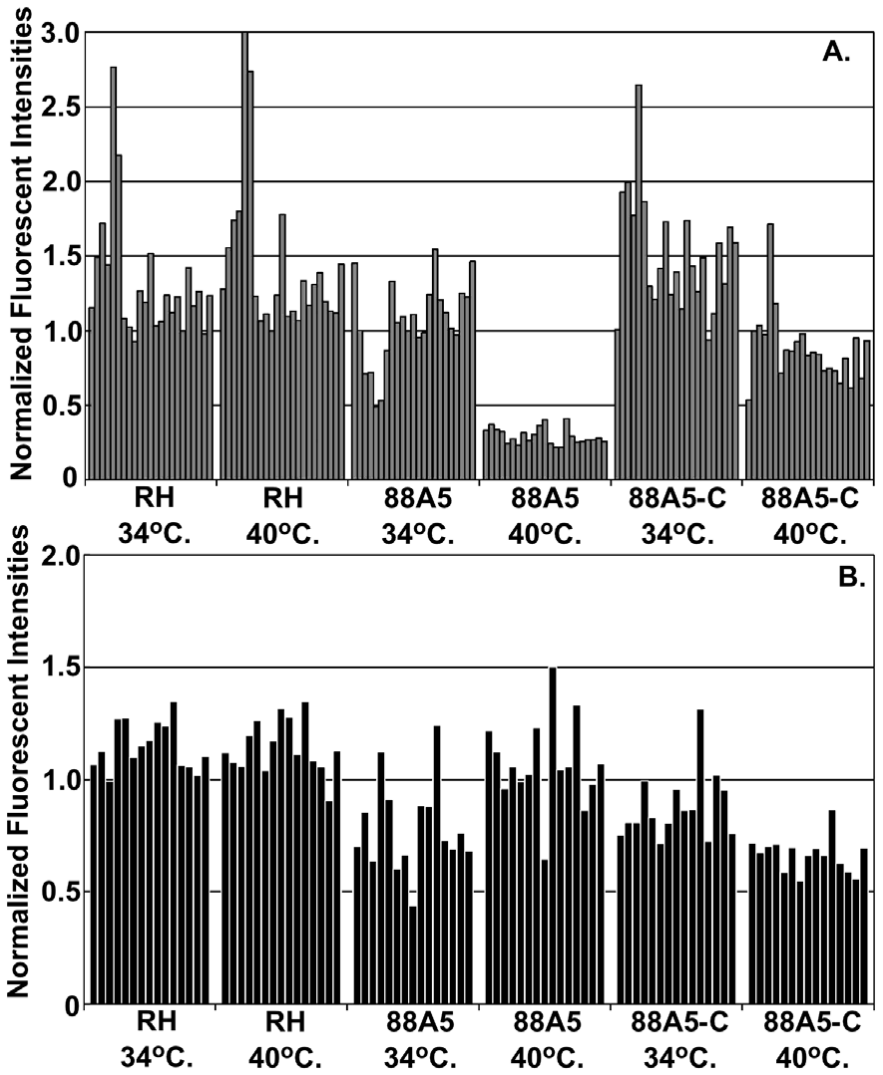
Dynamics of rhoptry mRNAs are linked to the early G1 period. Single point expression levels for rhoptry (**A**) and microneme (**B**) mRNAs with cyclic RNA dynamics were examined in a temperature sensitive mutant that growth arrests in the early G1 period of the tachyzoite cell cycle (mutant 88A5; [38]). RHΔ*hxgprt-* parent strain, ts mutant 88A5, and a genetically rescued 88A5 clone (88A5-C) were grown for 24 h at permissive (34°C) or non-permissive (40°C) temperatures. Duplicate RNAs were isolated and used to probe the *Toxoplasma* GeneChip. An expression heat map displaying the relative expression of all cell cycle genes in the mutant 88A5 and 88A5-complemented clone compared to the expression profiles of thymidine-synchronized tachyzoites is included in Figure S3. Normalized fluorescent intensities are shown for genes previously determined to have cyclic expression patterns (see Fig. 4E & G). Some variation (<2 fold) was detected between the parent and mutant strains in these experiments with rhoptry mRNAs having a larger range of abundance overall. However, the greatest difference in expression level observed was a consistent large decrease of the rhoptry mRNAs in mutant 88A5 parasites grown arrested at 40°C, which was rescued by genetic complementation.

Some of the largest amplitudes in the cell cycle transcriptome, and the steepest phases of net synthesis and decay, were shown by mRNAs of the rhoptry gene class (Fig. 4E). Genes with similar cyclical patterns defined a larger cluster that included mRNAs encoding IMC proteins (Fig. 4F), some kinesin and myosin proteins, and many hypothetical proteins (289 total). The timing of rhoptry mRNA increase followed by decay in the G1 phase of new parasites is consistent with the timing of *de novo* synthesis of these proteins in the previous mitotic period, and suggests that transcriptional as well as post-transcriptional mechanisms are coordinated in the parasite cell cycle. To further investigate the cell cycle timing of rhoptry mRNA levels, we examined whether the distinctive rapid decline of these transcripts would occur in a temperature sensitive growth mutant (88A5) that arrests in the early G1 period [38]. Total RNA was isolated in duplicate from the parental RH strain, from mutant 88A5, or from a genetically rescued 88A5 clone (88A5-C) all cultured for 24 h at the permissive (34°C) or non-permissive (40°C) temperatures. In previous studies, the phenotype of mutant 88A5 was classified in the G1 group based on cellular morphology (small parasites with single nucleus, no internal daughter forms) and the uniform haploid DNA content of growth-arrested populations [38]. A similar mRNA composition determined in these experiments also supports the assignment of this cell cycle arrest. In comparison to cyclical expression in the synchronized tachyzoite cycle, expression profiles of growth-arrested 88A5 parasites share similarities to tachyzoites progressing through the first half of G1 (Fig. S3 compare 6 h post-thymidine and 88A5-40°C), which included the distinctive steep decay of the rhoptry gene class (Fig. 6A). By contrast, rhoptry mRNA levels in genetically rescued 88A5 parasites at 40°C largely matches the average expression detected in randomly growing parasites at either temperature (Fig. 6A RH parent and 88A5-C, also Fig. S3). These results support a model whereby mechanisms active in early G1 parasites cause a rapid decline in the level of the rhoptry gene class mRNAs. That these changes are specific to mRNA class is evidenced by the absence of a significant decline in the levels of microneme mRNAs in all three parasite strains (Fig. 6B RH, 88A5, 88A5-C) grown at 40°C. Microneme mRNA profiles in synchronized parasites are offset in time (1–2 h) from rhoptry mRNAs (compare Fig. 4E to G) with minimum levels in mid-to-late G1, clearly past the point of cell cycle arrest in mutant 88A5.

### DNA motifs informed by cell cycle genes segregate between the major subtranscriptomes

**Figure 7:**
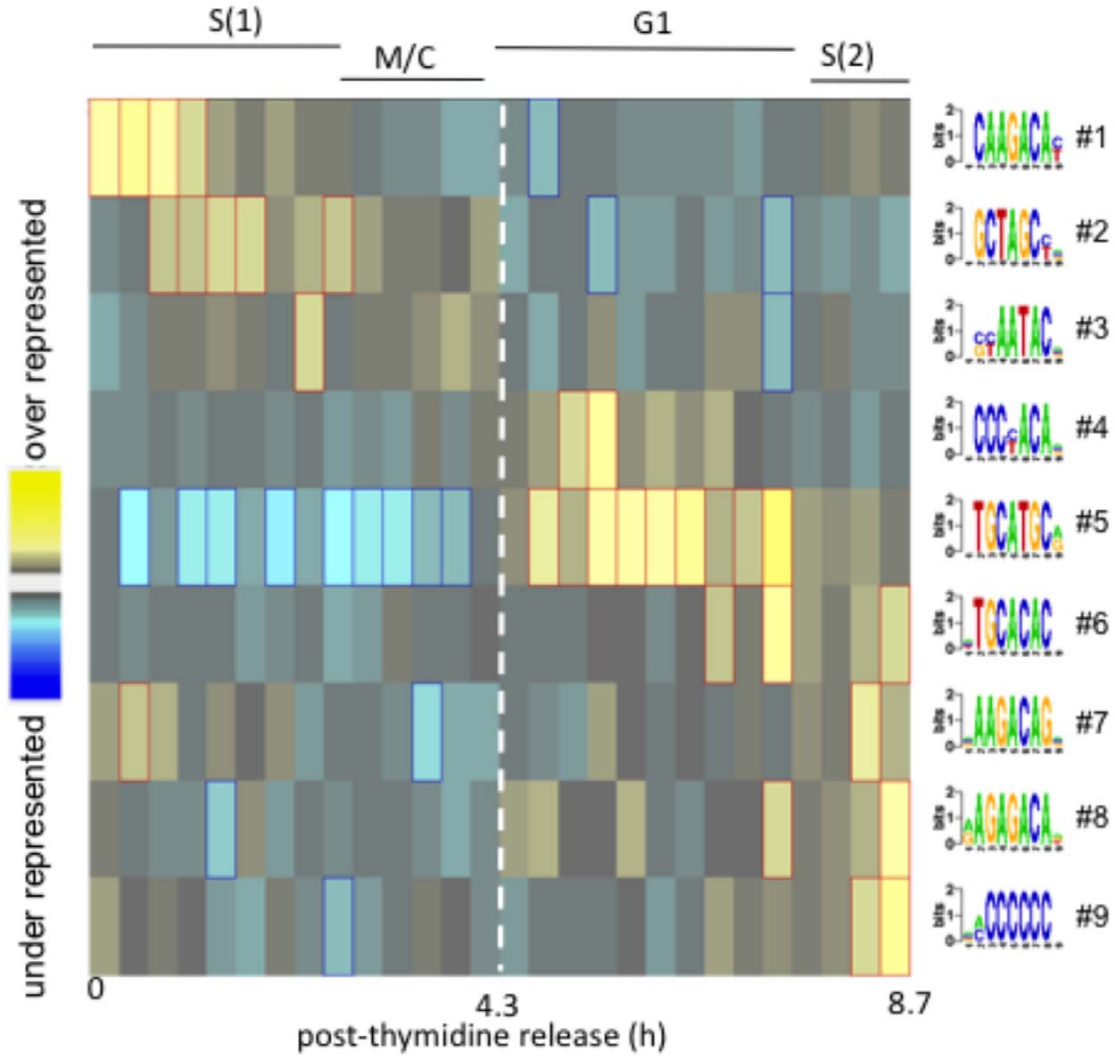
DNA motifs predicted by the cell cycle transcriptome. Over or underrepresented DNA motifs were identified in the 5′ regions of the 2,833 genes with cyclic mRNA profiles using the FIRE algorithm. For this plot, the intergenic regions interrogated were 0–2 kbp 5′-flanking to the coding regions, where the transcriptional start site predicted in ToxoDB (in some cases this is the ATG codon) was used to set 0 bp position. Intergenic regions were ordered by the peak time of mRNA abundance during the first complete tachyzoite cell cycle (identical order in Figs. 2A and Figs.3). Informative DNA motifs (rows) that are over or underrepresented in cohorts of similarly timed genes are shown (∼100 genes per vertical block; one complete cycle). No significant DNA motifs were detected if the expression order was randomized nor were DNA motifs found in the intergenic windows 2–4, 4–6, or 6–8 kbp 5′-flanking this gene set.

Computational approaches to identify DNA sequence motifs that regulate gene expression have not always achieved success due to widely different genome compositions and varying conservation of motifs functioning in gene promoters or RNA UTRs [39], [40]. Recently, a new computational method, FIRE (Finding Informative Regulatory Elements), which quantifies the dependency between sequence motifs and the continuous changes in gene expression was developed and has proven useful in finding stage-specific motifs in a range of organisms [41]. To investigate motifs associated with the parasite cell cycle transcriptome, we employed FIRE to interrogate four 2,000 bp intergenic regions (0–2, 2–4, 4–6, and 6–8 kbp) upstream of the coding regions of all 2,833 genes showing cyclic expression patterns. Intergenic regions paired with proximal cell cycle genes were ordered by the peak time of mRNA abundance during the first complete tachyzoite cell cycle (identical order in Fig. 3). FIRE analysis identified nine significant DNA motifs in the immediate 2,000 bp 5′-flanking region of cyclical genes (Fig 7) whereas only 0.04 motifs were predicted from 100 FIRE runs using randomly shuffled peak times. In contrast, genomic regions further upstream failed to yield either over or underrepresented motifs, which supports the proximal nature of *cis*-elements in experimentally mapped *Toxoplasma* promoters [42], [43], [44], [45], [46], [47]. The FIRE DNA motifs were found in gene promoters spanning the full time course of tachyzoite cyclical gene expression, remarkably resolving along the boundaries defined by peak expression, with DNA motifs overrepresented in one subtranscriptome generally underrepresented in the other (S/M versus G1). Motif-5 in particular was extensively overrepresented in hundreds of promoters flanking G1-subtranscriptome genes, while underrepresented in promoters flanking genes of the S/M-subtranscriptome. The sequence of motif-5 is identical to the TRP2 *cis*-element determined to be required for transcription of ribosomal protein genes [43], [48], and recently found to be widely dispersed in genomic sequences flanking more than 1,000 *Toxoplasma* genes [48]. Intriguingly, the TRP2 element is similar to the DNA binding site recently determined for the putative transcription factor AP2XI-3 (Gissot and Kim, personal communication), which had a peak expression in the G1 phase (Table S1).

### Serially expressed AP2 proteins are potential transcriptional specificity determinants throughout the cell cycle

**Figure 8:**
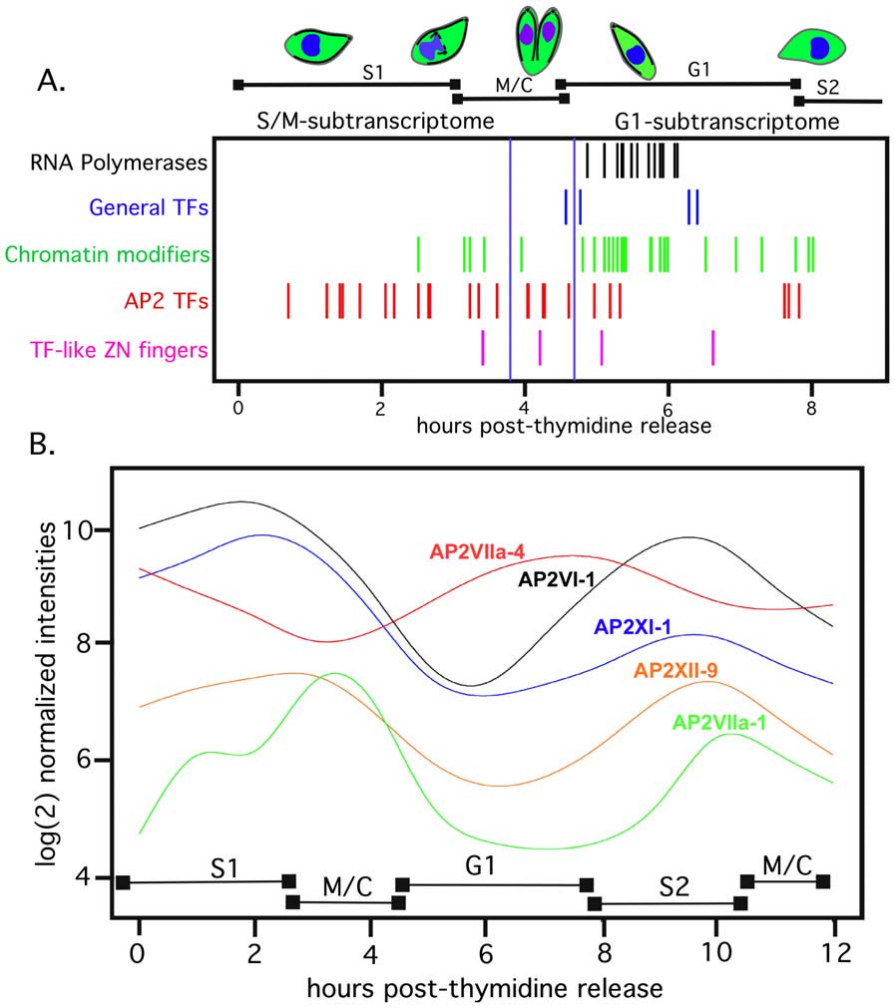
Putative transcription factors show cyclic RNA abundance patterns. Cell cycle expression profiles were assessed for 68 predicted AP2 domain genes [49] and 9 genes containing C2H2 Zn-finger domains (TGME49_103050, _121330, _101340, _086710, _006650, _002470, _059650, _023570, _111450) found in the *Toxoplasma* genome. (**A**) Maximum expression of RNA polymerases was tightly clustered in the G1-subtranscriptome along with a few general transcription factors. By contrast, the times of peak expression for 24 AP2 (see full list Table S1) and four activator Zn-finger domain genes were distributed across the tachyzoite division cycle (Table S2). In comparison, mRNAs encoding RNA polymerases showed peak expression clustered in G1. Relatively few general transcription factors and less than a third of known chromatin remodelers [87] also have cyclical patterns (see also Table S2). (**B**) Spline model curves for selected cell cycle AP2 mRNAs demonstrate relative abundance and time shifts indicating that the expression of these factors follows a serial order with peaks at different cell cycle stages.

The potential role of AP2XI-3 in the transcription of G1 mRNAs led us to explore the cell cycle expression of the complete AP2 family. More than seventy putative DNA binding proteins are recognized from the *Toxoplasma* genome; 68 genes contain one or more AP2 domains [49] while 9 genes (Fig. 8 legend) have the C2H2-Zn-domain of the type known to bind DNA in other eukaryotes [50]. In contrast to RNA polymerase genes that were co-expressed with peak mRNA times in a narrow window of early G1 (Fig. 8A, see also Figs. 3 & 4A), the *Toxoplasma* AP2 genes with cyclic profiles (24 of 68 total canonical AP2s, Table S1) showed distributed peak mRNA times (Fig. 8A). These 24 AP2 mRNAs varied in abundance and amplitude but together formed a time-order sequence that spanned the tachyzoite division cycle (see five example mRNA curves in Fig. 8B). Four out of the 9 mRNAs encoding C2H2-Zn-finger proteins also had cyclical patterns in tachyzoite division (Fig. 8A and Table S2), although in contrast with most of the cyclic AP2 mRNAs their profiles had very shallow amplitudes (not shown).

The serially expressed AP2 genes provide attractive candidates for transcriptional specificity determinants acting sequentially during *Toxoplasma* replication, although to advance this model the AP2 mRNA profiles across the parasite cell cycle must translate into ordered protein expression and translocation of AP2 factors to the nuclear transcriptional machinery. To answer this question for promising cell cycle TgAP2 factors, we utilized a new experimental method to tag the endogenous copy of *Toxoplasma* genes [51], [52]. In tachyzoites lacking the *ku80* gene (RHΔ*ku80*), recombination is predominantly at homologous sites allowing relatively small genomic fragments to direct the introduction of epitope tags into the endogenous locus of genes of interest. The RHΔ*ku80* strain was suitable for these studies because the growth rate as well as genomic DNA and cell cycle phase distributions were identical to the RH parent strain (Carruthers, Jerome, and White, unpublished results). C-terminal insertion of the YFP coding region by recombination into the endogenous locus was confirmed for five of the *Toxoplasma AP2* genes by diagnostic PCR of clone isolates (Fig. S3). The cell cycle stages at which the TgAP2yfp fusion proteins were expressed was determined by anti-IMC1 (daughters), anti-YFP (AP2s), and DAPI (genomic DNA) co-staining, employing criteria (cell size and marker expression) we previously established to pinpoint cell cycle arrest in a large collection of conditional mutants [38].

**Figure 9:**
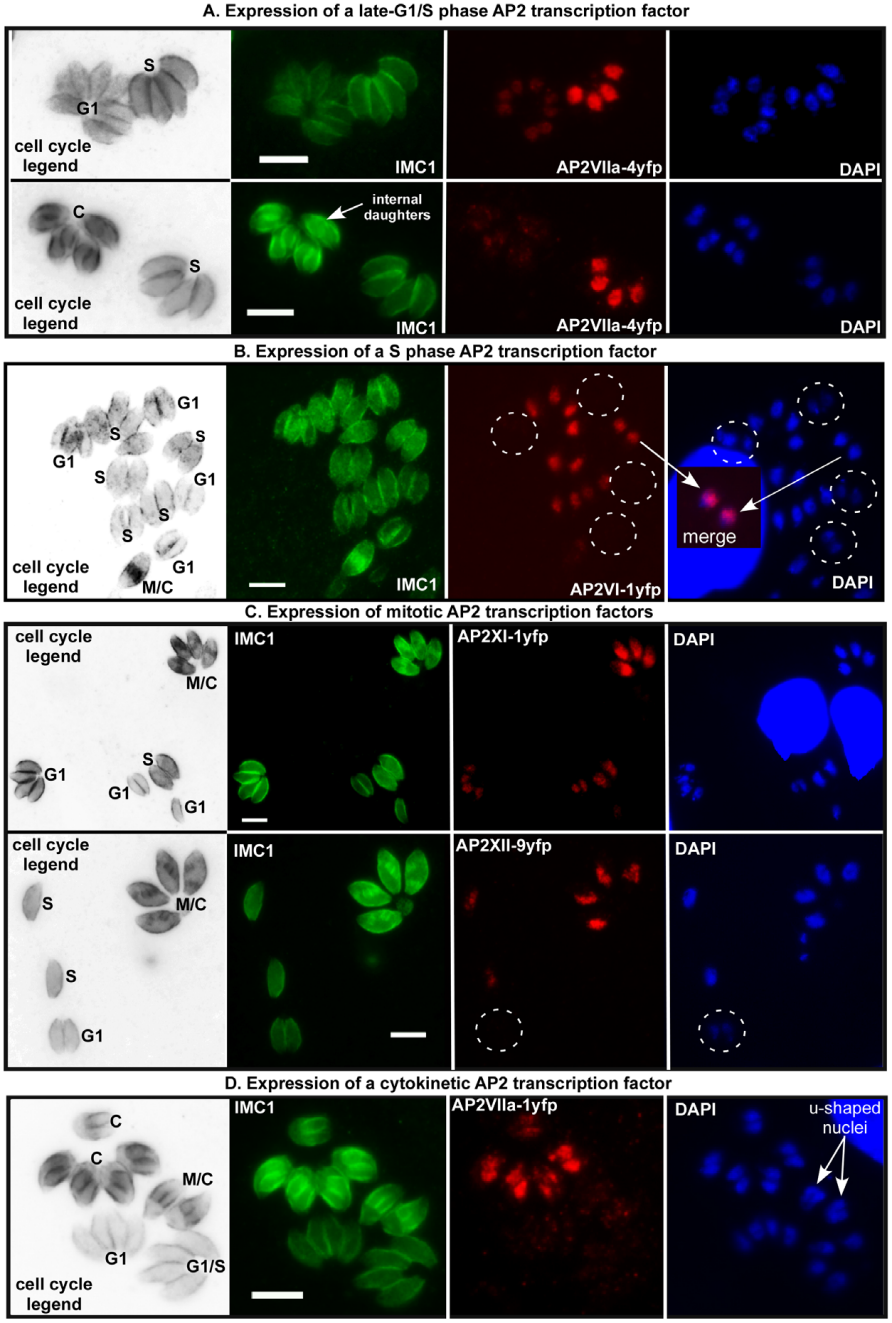
AP2 protein expression is coordinated with specific cell cycle phases. The endogenous loci of five cyclically expressed AP2 genes were epitope tagged with YFP in RHΔ*ku80* [51]. All tagged AP2 proteins were localized to the parasite nucleus. (**A**) AP2VIIa-4yfp was expressed beginning in G1 with peak expression in S phase (**B**) followed in time sequence by AP2VI-1yfp, which has an exclusive S phase expression profile. Merge inset indicates nuclear co-localization. (**C and D**) Three AP2 proteins had maximum expression in mitosis; AP2XI-1yfp and AP2XII-9yfp, while AP2VIIa-1yfp (**D**) was expressed in parasites that had divided their nucleus or were newly emergent from the mother. By mid-G1, AP2VIIa-1yfp was undetectable (not shown). Note; there is little expression of AP2VIIa-1yfp detected in parasites that were undergoing nuclear division (u-shaped nucleus) suggesting that the induction of this factor may be coupled to the completion of telophase. Co-stains: anti-IMC1 (green); anti-YFP (red), genomic DNA (DAPI, blue). Cell cycle legend is decolorized anti-IMC1 staining (inverted image) with labeling of internal daughters and cell cycle phases: G1 and S phase, C = cytokinesis, M = mitosis. Dashed circles denote the lack of AP2yfp expression, while the relative magnification is referenced by a 5 µm white bar.

*Toxoplasma* AP2yfp fusion proteins were localized to the tachyzoite nucleus with little evidence of regulated re-distribution between the cytoplasm and parasite nucleus for any factor (Fig. 9). All five tagged AP2 factors showed distinct peaks of protein expression following the order of their matched mRNA profiles shown in Fig. 8B. The increases and declines of these AP2yfp proteins mostly showed significantly steeper gradients than the corresponding post-thymidine mRNA profiles, reflecting possible mechanisms that act post-transcriptionally as well as the much tighter intravacuolar growth synchrony (relative synchrony windows; thymidine model ∼2 h versus intravacuole 10-20 min). Fusion protein AP2VIIa-4yfp was first detected in G1 parasites, although the peak expression was in S phase (Fig. 9A, top images). AP2VIIa-4yfp declined to nearly undetectable levels in mitotic/cytokinetic parasites that contain internal daughter forms (Fig. 9A example in bottom images). AP2VI-1yfp was exclusively expressed in S phase parasites that were distinguished from G1 parasites by their larger cell and nuclear sizes and increased DAPI staining. The representative vacuoles shown in Figure 9B provide examples of five positive S phase and four negative G1 vacuoles. Like AP2VIIa-4yfp, levels of AP2VI-1yfp fusion protein declined rapidly in parasites possessing early internal daughter forms indicating that as parasites exit S phase cell cycle mechanisms quickly reduce the levels of this protein. By contrast, three AP2yfp fusion proteins had peak expression in parasites containing internal daughters. We observed high levels of AP2XI-1yfp and AP2XII-9yfp (Fig. 9C) in tachyzoites that contained daughter forms at all stages of maturity, which is a pattern consistent with parasites undergoing the mitosis through nuclear division (daughter buds first form in late S phase, [37], [53]). AP2XI-1yfp differed from AP2XII-9yfp, in that it was detectable throughout the tachyzoite cycle, although in G1 parasites there was a significant decline in protein level (Fig. 9C, top panel). Perhaps the most exclusive expression pattern belonged to AP2VIIa-1yfp (Fig. 9D). Expression of AP2VIIa-1yfp was confined to parasites in late cytokinesis that extended into newly emergent parasites in early G1 (not shown). This fusion protein showed a narrow peak during the late stages of the tachyzoite cycle with elevated levels tightly coincident with the completion of nuclear division; a remarkable timing represented by the adjacent parasite vacuoles of Figure 9D, whose vacuolar synchronization differs by only a few minutes in the late mitotic/cytokinetic processes (the parasite vacuole with u-shaped nuclei is negative, while the second vacuole with divided nuclei is positive). Altogether this set of AP2yfp fusion proteins comprised an overlapping series with the potential to mediate successive periods of specific promoter occupancy and transcriptional activation, with the first AP2yfp fusion protein appearing in mid to late G1 and the last protein disappearing as new daughter parasites entered the next cell cycle (Fig. 9, cell cycle expression order AP2VIIa-4→AP2VI-1→AP2XI-1 = AP2XII-9→AP2VIIa-1).

## Discussion

Apicomplexan parasites replicate by multiple strategies that differ in the number of nuclear divisions per cycle, although all share the process of forming new daughter parasites by internal budding. Understanding the molecular basis of these unusual processes is desirable for the potential new therapies this knowledge might yield. Utilizing a thymidine-synchronization model, we have analyzed the cell cycle changes in the profiles of tachyzoite mRNAs that accompany nearly two cycles of tachyzoite division. The cell cycle transcriptome that emerged implicates ∼40% of the 7,784 coding sequences assayed by microarray and is organized in two major transcriptional waves bounded by the commitment to DNA synthesis and cell cycle exit. Our mathematical analyses served to minimize artifacts that may have led to overestimates of the number of cell cycling genes in synchronized yeast models [29], [30], which suggest that 24–29% of budding yeast genes have cyclic patterns (significantly fewer than we find in *Toxoplasma*). Our cubic B-spline methods used to model the cell cycle time courses, together with PCA and SVD analyses of the mRNA data matrix across all genes and time points, provided objective criteria for identifying cyclic mRNA patterns and the natural grouping of genes into two predominant subtranscriptomes.

**Figure 10:**
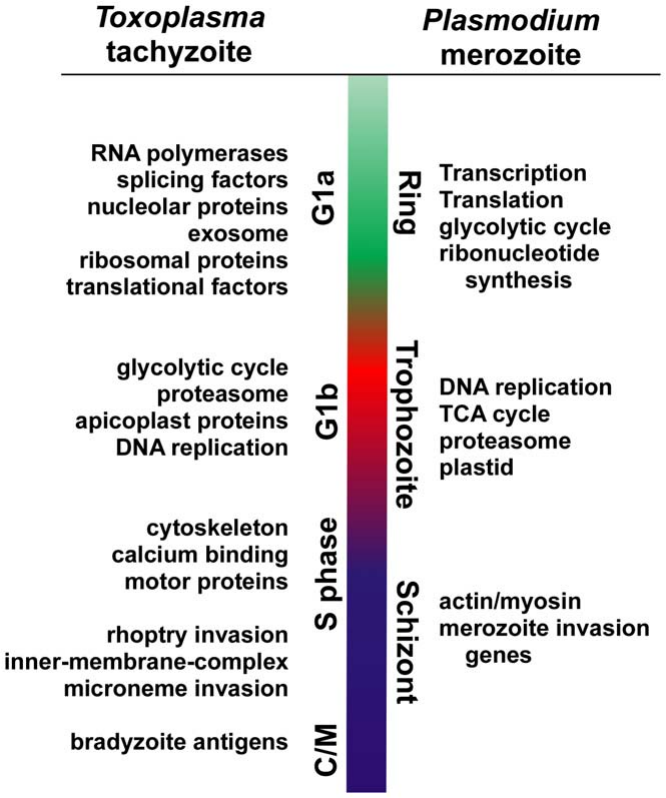
Gene expression during asexual replication of *Plasmodium* merozoites and *Toxoplasma* tachyzoites follows a similar gene order.

The specific genes ordered within each wave by peak expression provide insight into the widely conserved processes and the specifically apicomplexan evolutionary innovations active in *Toxoplasma* cellular growth and division, which are generally shared with dividing merozoites of *Plasmodium* (Fig. 10). The timing of protein and DNA synthetic components and central metabolic functions in the early and late tachyzoite G1 period and *Plasmodium* merozoite ring and early trophozoite stages [13], [14] follows a similar sequence in other eukaryote cell cycles [54], [55], [56] suggesting this program of gene expression has a long evolutionary history. The classic partition of G1 into two subcompartments (a and b) with two different states of mRNA content (low versus high) in the G1 period of mammalian cells [57] has parallels to the low levels of RNA synthesis in the *Plasmodium* ring stage that quickly increases with progression to early trophozoites [58], [59], [60]. This change in RNA is conditional on the status of ribosome biogenesis, which cells monitor to gauge the nutrient support for protein translation [61], prior to committing to DNA synthesis (START checkpoint). The cell cycle mechanisms that sense the status of ribosomes/translation factors [63], [64] are interconnected to the induction of genes required for DNA replication in late-G1/S through G1 cyclin regulation. These mechanisms follow the same regulatory logic in yeasts and multicellular eukaryotes but the detailed protein interactions are different. In metazoans, G1 coordination involves retinoblastoma protein, E2F transcription factor and cyclin D & E (reviewed in [65]), while in yeast the SBF/MBF transcription complexes and cyclins CLN1-3 are the key factors (reviewed in [66]). There are few details of how cyclin/cdk pathways work in apicomplexans and orthologs for many of the known G1 regulators appear to be absent based on preliminary genome surveys, although some cdk homologs with unknown substrates are present. Nonetheless, evidence for checkpoint mechanisms in the parasite G1 phase have surfaced in studies of conditional growth mutants [38] and in the discovery that tachyzoites can be reversibly arrested in G1 [25]. Another intriguing possibility for coordinated G1 transcriptional activation has now emerged from our FIRE analysis (Fig. 7) in which the principal G1-associated DNA motif 5′-TGCATGC is a known *cis-*element present in hundreds of *Toxoplasma* promoters [43] and recognized by a conserved G1 (ring stage) specific AP2 factor in the *Plasmodium falciparum* cycle [67].

Unlike animal cell division, which results from fission of the mother into two daughters, new *Toxoplasma* tachyzoites are assembled internally and their growth consumes the mother [7], [68], [69]. The timing of this unique process begins with the appearance of conoids in late S phase [8] and culminates with fully formed daughters possessing a single nucleus and full complement of organelles; *e.g.* plastid, rhoptry, and micronemes [35], [37]. The biosynthetic demands of budding are likely considerable, which along with the need to coordinate budding with nuclear division [8], consigned the building new daughters and their invasion apparatus to the second half of the parasite cell cycle. Consistent with this biology, tachyzoite commitment to DNA replication was associated with a rapid rise in large number of apicomplexan specific mRNAs in the subsequent S-M periods. These specialized mRNAs in *Toxoplasma* are abundant and collectively dominate the tachyzoite mRNA pool [70]. Gene expression in the *Toxoplasma* S/M-subtranscriptome is influenced by the functional requirements of daughter formation. Thus, specialized genes encoding secretory products and components of the unique parasite cytoskeleton are co-expressed with others encoding motor proteins and many signal transduction factors necessary for complex assembly. The mRNAs encoding proteins destined for *Toxoplasma* rhoptry and microneme apical organelles are expressed in the precise order of their biosynthetic construction (rhoptries before micronemes; [35]) and each mRNA class could be further resolved into gene subsets that have distinct expression profiles (Wootton, Behnke, and White, unpublished). Whether this signifies multiple transcriptional mechanisms act in series to specify activation and timing of each major organelle protein class is unknown. However, native promoter recognition is clearly important for organelle biogenesis since altering the promoter context disrupts protein targeting (*e.g.* microneme proteins *T. gondii* TgSUB1 or *Plasmodium* AMA-1; [71], [72]). Over expression was offered to explain the mis-targeting of these proteins [72], although the cell cycle phase specific transcription of these genes was unknown at the time of these studies. Given our current knowledge, it is now plausible that the failure to reach the appropriate organelle compartment may have been caused by missing the delivery window required for new organelle production. In this regard, mechanisms mediating both synthesis and decay of mRNA levels by the exosome complex may be equally important for expression timing of invasion genes as is clear from our studies of the mRNA and protein profiles of organelle proteins in a putative checkpoint mutant with early G1-arrest (Fig. 6).

In budding yeast, the intersection of checkpoint mechanisms at strategic points along the cell cycle continuum is believed to foster coupled serial interactions between certain transcription factors and regulate the pace of transcriptional change through key cell cycle transitions (see reviews [73], [74]). It is unknown whether this type of transcriptional regulation may operate in the peculiar and varied replication schemes used by apicomplexan parasites to reproduce. Until recently it would have been difficult to envision mechanisms of serial transcriptional activation in the Apicomplexa. However, the discovery of multiple plant-like AP2 transcription factors [21] in these parasites now offers a source of transcriptional regulators upon which to construct such a process. A protein interactome map of *P. falciparum* recently showed several AP2 proteins interacting with the epigenetic machinery, including GCN5 histone acetyltransferases and SWI2/SNF2 ATPases [75], consistent with a role in transcriptional activation [76]. De Silva *et al.* [67] used protein-binding microarrays to elucidate DNA binding motifs for select *Plasmodium* ApiAP2 factors; the motifs were subsequently found as *cis-*elements in gene groups that are coordinately expressed. It was no surprise then that functional validation of an AP2 factor was demonstrated in recent studies, which established the essential roles for *Plasmodium berghei* AP2 factors in the formation of ookinete and sporozoite developmental stages [77], [78]. In addition to transcriptional activation, AP2 factors may act as suppressors of gene expression or have roles in regulating chromatin structure [79], [80]

The apicomplexan AP2 domains are related to plant AP2 factors and have likely evolved as a major lineage specific expansion within the Alveolata from a single progenitor [21]. The *Toxoplasma* genome encodes a large number of proteins (68 total) with one or more closely related AP2 domains (see Table S1 and [49]); more than twice the number found in *Plasmodium falciparum* (29 total, [49]) and other apicomplexans [21]. In our study, a third of *Toxoplasma* AP2 genes (24 total) showed cell cycle profiles with peak expression times distributed throughout the tachyzoite cell cycle. Surveys of other microarray data in *Toxoplasma* has identified 11 *Toxoplasma* AP2 mRNAs induced during bradyzoite differentiation, 27 are constitutively expressed, and 6 show no expression in the intermediate life cycle stages indicating they may have roles in other stages of development (Table S1). Cell cycle and bradyzoite developmental AP2s are largely separate gene groups with the exception of AP2VIIa-1, which has peak expression in tachyzoite cytokinesis and is also substantially increased under bradyzoite induction conditions *in vitro* (Behnke and White, unpublished). Previous studies by our group have pointed to the late periods of the tachyzoite cell cycle as important to bradyzoite differentiation [81]. Intriguingly, bradyzoite mRNAs that had cyclical profiles reached maximum levels in late cytokinesis, which is similar timing to this potential dual function AP2 factor.

We have shown here that five of the cell cycle AP2 factors had sharp peaks of protein expression in the same restricted cell cycle timeframes as their mRNA patterns and were exclusively nuclear in the tachyzoite. The serial expression of as many as 24 AP2 mRNAs and proteins immediately suggests a scenario of sequential transcriptional modulation mediated by AP2 domains with different promoter recognition specificities through the cell cycle that could be required for parasite replication. Consistent with this model we have found that deletion of specific AP2 factors is either not tolerated or in some cases leads to mutants with significant cell cycle defects (Radke, Lucas, and White, unpublished). It is not yet known if AP2 factors may themselves be subject to these cell cycle transcriptional mechanisms; *i.e.* induced AP2 proteins may be part of the regulatory logic and interactions that determine the next factor(s) in the time order. Such regulation would be consistent with mechanisms involving both checkpoint transitions and transcriptional regulation as proposed for cell cycle progression in other eukaryotes (see reviews [73], [82]). Investigating these relationships in *Toxoplasma* will be important for understanding the balance of regulatory links that ensure the fidelity of parasite growth.

## Materials and Methods

### Parasite culture and synchrony model

Parasites were grown in human foreskin fibroblasts (HFF, which were kindly provided by David Roos (University Pennsylvania, see ref. [83]). All transgenic and mutant parasite lines are derivatives of the RH parasite line: the RH^TK+^ strain expresses the fusion protein of herpes simplex thymidine kinase and chloramphenicol acetyltransferase [23]; temperature sensitive clone 88A5 was obtained by chemical mutagenesis of the RHΔ*hxgprt-* strain [38]; RHΔ*ku80* strain [51] was utilized for direct tagging of AP2 genes with YFP.

Synchronization of RH^TK+^ parasites with thymidine followed published protocols [23]. Briefly, parasites were seeded at an MOI of 1:1 and grown for 12 h at 37°C (4–8 parasites per vacuole). To block RH^TK+^ growth, overnight culture media was replaced with pre-warmed DMEM media containing 1% fetal bovine serum and 10 µM thymidine. The flasks were incubated for 4 h at 37°C and then quickly washed 2x with pre-warmed DMEM with the final wash replaced with pre-warmed DMEM media containing 1% serum. RH^TK+^ parasites from multiple T175cm^2^ flasks (10–20 per RNA sample) were purified from host cells for RNA isolation as previously described [83]. Each duplicate parasite sample for RNA isolation of temperature sensitive mutant 88A5, RHΔ*hxgprt-* parent, or a genetically complemented 88A5 clone was obtained by combining three T175 cm^2^ flasks (MOI 1:1) grown for 24 h at 34°C. Three infected flasks were also combined for the RHΔ*hxgprt-* parent and genetically complemented 88A5 clone grown at 40°C. Due the quick arrest of mutant 88A5, 25 flasks (T175 cm^2^) were combined in order to isolate sufficient RNA for microarray hybridization from the mutant grown at 40°C.

Nuclear DNA content of synchronized RH^TK+^ parasites was evaluated by flow cytometry using SYTOX Green (Invitrogen) staining of tachyzoites [22]. Representative samples from pooled populations purified for RNA isolation were collected by centrifugation and resuspended in 300 µl cold PBS and 700 µl of cold 100% ethanol added drop-wise. The fixed samples were stored at −20°C for at least 24 h prior to staining for flow cytometry. Fixed parasites are pelleted at 3000×*g*, resuspended in 50 µM Tris pH 7.5 at a final concentration of 6×10^6^ parasites/ml and stained with SYTOX Green (1 µM). RNase cocktail (250 U; RNase A, RNase T1) was added and the parasites incubated in the dark at room temperature for 30 min. Nuclear DNA content was measured based on fluorescence (FL-1) using a 488 nm argon laser flow cytometer. Fluorescence was collected in linear mode (10,000 events) and the results were quantified using CELLQuest™ v3.0 (Becton-Dickinson Inc.). Staining of RH^TK+^ infected cultures with a monoclonal mouse antibody 45.15 for IMC1 (1:2000, a gift from Gary Ward, University of Vermont) was used to examine changes in cell morphologies and to determine the fraction of internal daughter buds. Briefly, parasite cultures grown in parallel on coverslips in 6-well plates were fixed with 3.7% paraformaldehyde (pH7.4) and then permeabilized with 0.25% Triton X-100. Coverslips were washed and incubated in 1X PBS pH 7.4 containing 5% FBS and 3% BSA (blocking solution) for at least 30 min. Primary antibody diluted in blocking solution was incubated for 30–60 min, the coverslips washed 3X with blocking buffer and then incubated for 30–60 min with secondary antibodies: Alexa Fluor 594 for mouse diluted 1:2000 in blocking buffer. The secondary antibody was removed and the coverslips mounted in gel mount. Parasites were evaluated with an epifluorescence microscope (Eclipse TE300, Nikon Inc., Melville NY) and images collected with a digital camera (SPOT™, Dynamic Instruments Inc).

### Microarray hybridization and statistical analysis

RNA was extracted from parasites using the RNeasy kit with β-mercaptoethanol and DNase I treatment (Qiagen, Valencia, CA). RNA quality was determined using the Agilent Bioanalyzer 2100 (Santa Clara, CA). A total of 3 µg starting RNA was used to produce cRNA using the Affymetrix One-Cycle Kit (Affymetrix, Santa Clara CA). Fragmented cRNA (5 µg) was hybridized to the *Toxoplasma gondii* Affymetrix microarray (ToxoGeneChip: http://ancillary.toxodb.org/docs/Array-Tutorial.html) according to standard hybridization protocols. Two hybridizations were done for each sample type and all data were deposited at NCBI GEO (GSE19092). Hybridization data was preprocessed with Robust Multi-array Average (RMA), normalized using per chip and per gene median polishing and analyzed using the software package GeneSpring 7.2 (Agilent Technologies, Santa Clara CA). An ANOVA was run in order to identify genes with significantly greater than random variation in RNA abundance across the synchronized cell cycle data grouped by hour from blocked (R0) to 7 hr (R7). Variances were calculated using cross-gene error model, with a p-value cutoff 0.1 (FDR.1), and multiple testing correction: Benjamini and Hochberg False Discovery Rate. This restriction tested 8,131 probe sets; 43 genes had insufficient data for a comparison. About 10.0% of the 3,518 probe sets identified would be expected to pass the variance restriction by chance.

Principal components analysis (PCA) was carried out by conventional methods using the eigen() function of Splus or R, starting with (a) the 2,833×2,833 between genes covariance matrix and (b) the 14×14 between samples covariance matrix (13 time points plus the asynchronous control). For the co-plot of the 2-dimensional projections from both (a) and (b) (Fig. 2B), a single arbitrary scaling factor was applied to eigenvectors 1 and 2 of (b) to enable the plots to be compared visually while maintaining the original zero points and aspect ratio.

Matrix singular value decomposition (SVD) was carried out by conventional methods by applying the svd() function of Splus or R to the 2,833×13 (cyclic genes) and the 7,784×13 (all genes) matrices of genes by time-points. Results were interpreted following the principles established for budding yeast cell cycle studies by Alter and coworkers [31], [84], as described in Fig. 2C and the related main text. The principal eigenvalues from SVD also provided estimates of the fractions of the total mRNA amounts (aggregated over either the 2,833 cyclic genes or all 7,784 genes) attributable to the non-cyclic steady-state expression level present throughout the cell cycle and to the superimposed cyclically varying components of the two subtranscriptomes. For this purpose, an additional step of all-gene normalization, as applied to non-spiked controls by [85], was applied to the hybridization intensity data (after RMA normalization, log(2) transformation, and baseline subtraction), although this made only small (4.6% or less) differences to the 13 time point values consistent with near constancy of RNA input amounts. These all-gene normalized values can be interpreted as indicators of mRNA abundances since c. 90% of the genes with cyclic patterns fall in the linear dynamic range (compare [85]).

All microarray data presented in this study is scheduled for upload and presentation by individual gene and time course at the genome web site, ToxoDB, which is part of EuPathDB (http://eupathdb.org/eupathdb/). These data have been deposited at GEO with accession GSE19092.

### Spline models of expression profiles

To estimate expression profiles as continuous functions over time, we used cubic B-spline smoothing models, following methods previously established for microarray data [26], [27], [28] with the modifications described below and in Fig. S2. Our analysis had three goals: first, to serve as an automated computational procedure to distinguish cell cycle related profiles from those with arbitrary or insignificant curvature; second, to estimate from each model profile the times of maximum and minimum RNA abundance and the times of maximum rates of RNA increase and decrease; third, to identify sets of genes with similar expression profiles. For these purposes we needed a robust estimate of each individual gene expression profile from the time series of 13 data points and sufficiently stringent error analyses to support subsequent profile neighboring and clustering. We have not iteratively refined the profile models of individual genes using consensus models from clusters of biologically related genes as previously done [26], [27], [28]. Instead, we have used an empirical error analysis to evaluate the confidence that can be placed in each individual model given its supporting microarray data, thus reducing prior bias and avoiding circular reasoning when genes are subsequently compared by profile neighboring.

The core procedure used to fit a cubic B-spline model to each series of 13 data points was the smooth.spline() algorithm in S-Plus or R (the mathematical basis can be found in [26], [27], [28]). Preliminary experiments varying the granularity of smoothing using the *spar* and *all.knots* options of smooth.spline(), revealed a useful property of this algorithm, namely that only 3 clearly distinct classes of spline models, which we call ‘phases’, were produced from the data. These can be characterized by curve shape, as follows (examples in Fig. S2A): (1) ‘Single-wave’, typically with just one peak/trough per 8 hour cycle, as shown by the large majority of genes for which the data show an unambiguous cell cycle related pattern, and also others; (2) ‘Double-wave’, typically showing doublet peaks or peaks/troughs determined only by single data points, usually cases of over-fitting; (3) ‘Linear’, with a straight line fit across the entire time series, as with the large majority of genes excluded as cyclic candidates by the variance criterion. Consequently, a key question for error analysis, and for assignment of profile patterns as cyclic or non-cyclic, is whether the phase of spline model computed from the 13 data points is robust and truly represents the underlying RNA dynamics. Robustness requires a strongly determined phase that is not readily tipped into one of the other two phases by minor data variation within the usual range of error. This question is not addressed by the conventional computation of 95% confidence intervals from the residuals of the spline model regression on the data points since such intervals are constrained to the original phase of the model. Accordingly, we implemented the following empirical test of phase robustness based on simulated errors and thresholds; these were chosen conservatively to avoid over-assigning profiles patterns as cyclic. For each 13-point time series, 1,000 variants of the data were generated each with independently-seeded random uniform noise added/subtracted onto all 13 numbers (log(2) scale) and bounded within a +/− 10% range of each number (where 100% is the span of the 13 original numbers). The following code implements the core routine, which takes as input a 13-point vector ‘(gene.data)’, applies the data perturbation, and produces a matrix of the 1,000 simulated spline curves (‘variant.splines.matrix’) for further analysis or plotting (examples in Fig. S2A):

variant.splines.1000<− function(gene.data,x) {variant.splines.matrix <− matrix(nrow = 1000,ncol = x) for(i in 1:1000) {variant.splines.matrix[i,] <− spline(smooth.spline(jitter(gene.data,5)),n = x)[[2]] } variant.splines.matrix}

Here, the function jitter() implements the runif() function for generating random uniform noise with the parameter 5 defining the +/− 10% limits described above. The parameter *spar* of smooth.spline() defaults to zero which determines that the smoothing parameter for each variant spline curve is selected by cross-validation; this selection produces models that are favorable for later phase classification as Single-wave, Double-wave, or Linear by an automatic variance ratio test. The function spline() interpolates *x* points (with no further smoothing) into the model previously fitted by smooth.spline(): these points provide any ‘pixel granularity’ needed for visualization of the cubic B-spline models as smooth curves, *e.g.* as in Fig S2A.

The rules for assigning a profile pattern as cyclic or non-cyclic were based on the distribution of phases in the ensemble of the 1,001 spline models (1,000 error simulations plus the model from the real data), as follows: (1) if the model from the real data was Linear, or if more than 4 error simulated models were Linear, the assignment was ‘non-cyclic’ (examples: the blue ensembles in Fig. S2A; the black lines in Fig. S2B); (2) in other cases, the assignment was ‘provisionally cyclic’ (the proviso being that the cell cycle related peak/trough recurrence criterion had not been applied at this stage), and the phase of the model was classified as Single-wave or Double-wave according to the model from the real data. The large majority of provisionally cyclic models were assigned as Single-wave type by this rule (colored red in Fig. S2A and S2B), although many of these showed a mixture of Single-wave and Double-wave phases in the error simulations. Double-wave profiles were assigned by rule (2) in a small minority of cases (example: the green curve labeled ‘AP2VIIa-1’ in Fig. 8); these doublets may reflect over-fitting rather than biological reality but we report them as they stand following the principle of fidelity to the data.

From each spline model curve, the times of maximum and minimum RNA abundance, and the times of the inflexion points corresponding to maximum rates of RNA net increase and decrease, were computed by conventional second derivative techniques using the peaks() function of Splus with local span lengths 1/40 of the total profile length. The same computation was applied to the 1000 simulated error curves and used to estimate peak time standard errors (Table S1) after excluding any error curves with gross phase differences from the real data model. We then tested if the recurrence interval between peaks or troughs within the first and second cell cycle windows was in the range 7.4-8.7 hours. The blue lines in Fig. S2B are examples of ‘provisionally cyclic’ profiles that were excluded by the recurrence interval rule from the final list of 2,833 genes with canonical cyclic expression patterns.

### Finding Informative Regulatory Elements (FIRE) analysis

Gene proximal sequence, 2000 bp 5′ and 100 bp 3′ of the transcriptional start site obtained at ToxoDB.org [86], and peak mRNA ordered gene expression information was used in combination with the FIRE computational tools [40] to identify possible over or underrepresented *cis-*motifs. Several controls were conducted by running 100 permutations of randomly ordered max mRNA peaks, as well as using sequence further upstream from the TSS (4000-2000 bp 5′, 6000-4000 bp 5′ and 8000-6000 bp 5′ from TSS). No significant motifs were found in any of these upstream regions. Default parameters were used on all runs of the continuous datasets.

### Western analysis of synchronized parasites

Following synchronization of parasite growth by thymidine block, cells were harvested at R0, R2, R4 and R6 time points and frozen as pellets at −80°C. Cells were resuspended in Laemmli sample buffer containing protease inhibitors (10 mg/ml E-64, 100 mg/ml AEBSF, 100 mg/ml TLCK, 10 mg/ml leupeptin (Sigma-Aldrich, St. Louis, MO) and boiled for 5 min. Lysates were reduced with addition of 2% β-mercaptoethanol except for blots performed with anti-SAG1 and anti-AMA1 antibodies as these are sensitive to reduction. A total of 5×10^6^ cells were loaded per lane and resolved on 10% PAGE gels. Gels were transferred to nitrocellulose membranes and blocked overnight at 4°C in blocking buffer (PBS, 0.05% Tween-20, 10% normal goat serum, 0.05% non-fat dry milk). Membranes were incubated with the following antibodies diluted in blocking buffer: anti-ROP2 (Rab249) at 1:2000, anti-Actin (ACT1) (RabMO372) at 1:5000, anti-β-Tubulin (b-TUB) (RabWU1430) at 1:5000, anti-MyoA (Rab 113d, courtesy of Dominique Soldati) at 1:1000, anti-MIC2 (mAb 6D10) at 1:10000, anti-IMC1 (mAb 45.15, courtesy of Gary Ward) at 1:10000, Anti-AMA1 (mAb B3.90, courtesy of Gary Ward) at 1:8000, or anti-SAG1 (mAb DG52) at 1:20000. Following washing in PBS 0.05% Tween-20, membranes were incubated with goat anti-mouse IgG or goat anti-rabbit IgG conjugated to HRP (Jackson ImmunoResearch Laboratories, Inc., West Grove, PA). Membranes were washed and incubated with ECLplus Western Blotting Detection System (GE Healthcare, Piscataway, NJ) and imaged using a FLA500 Phosphorimager (FujiFilm Medical systems, Stamford, CT). The experiment was repeated three times with similar results and representative quantification of signals adjusted for background are shown in Figure 5.

### YFP tagging of AP2 genetic loci

Genomic fragments were amplified by PCR for each cell cycle AP2 gene using a design that fuses the C-terminal end of each coding region with YFP in vector pYFP.LIC.DHFR [51]. The PCR designs partially covered the coding regions, and therefore successful expression required recombination into a locus supplying the appropriate translational start context. Primer designs: AP2VIIa-4 forward 5′-CCTATCGCCGACCTCTCTGGCTGCAGAC-3′, reverse 5′-GTCCGTTCTGGCCTTTTTGCTAGACGAAGCCGCC-3′; AP2VI-1 forward 5′-CGCAAACATCCAGTTCATCGGTATGC-3′, reverse 5′-CGCGTGGGCCGCTGATACTTCAGCAACCGC-3′; AP2XI-1 forward 5′-GCACACGCGAGCTGGATCCGAGCGCTG-3′, reverse 5′-GCACCACTCAAACTCCTGACGTGGAAC-3′; AP2XII-9 forward 5′-GCGCGGGCAGCTTGCGAAACCGTCTTG-3′, reverse 5′-GACGTCCAGAAAAGACTTTTCATGTTCTTCGAATGCCG-3′; AP2VIIa-1 forward 5′-GCGACGACATGTTGCCACGACGTCGGAGC-3′, reverse 5′-CCGGCCTCGTCGCCGCCAATCCACGC-3′.

PCR fragments were cloned by a ligation-independent method as described [51]. In brief, T4 DNA polymerase was used to generate single stranded ends in both fragment and vector, the DNAs hybridized and the mixture transformed into *E. coli*. The resulting tagging constructs were electroporated into the RHΔ*ku80* strain and stable transformants selected in media containing 1 µM pyrimethamine. Cloned isolates were screened to verify homologous recombination (Fig. S4) using a unique 5′-primer upstream of each genomic fragment used to tag the locus in combination with a common YFP primer or a unique 3′-UTR primer to confirm loss of the wild type locus (see Fig. S4 for screening design and results). Genomic DNA was isolated from YFP positive clones and compared to RHΔ*ku80* parent DNA. Screening primer designs: common reverse YFP, 5′-GTCGATGCCCTTCAGCTCGATGCGG-3′; AP2VIIa-4 screening forward 5′-TGCTGGCACCGCCAACGGCACTAC-3′, screening reverse 5′-AATCGGCGTACGATGAGAGGCAATGC-3′; AP2VI-1 forward 5′-GGTGAGAAGCAAGAGGCCACAGAGGCC-3′, reverse 5′-GCATGCCAAAGAACGCCGCCCTCGACC-3′; AP2XI-1 forward 5′-GCGAAGTGAACGCGGCGAGGCGAGG-3′, reverse 5′-CTCCCATCGCTCCTCATGTCCGTCTACGG-3′; AP2XII-6 forward 5′-AGTGTGTGGAGACTCGCTCGTGA-3′, reverse 5′-ATTGGAGATTGAGCGACCCCAGACTG-3′; AP2VIIa-1 forward 5′-AATAGAGGCGAGGAGTGTTGCAGTC-3′, reverse 5′-ATTCGCTGCTGCGTTGGCCTCGA-3′. AP2yfp tagged clones were subjected to immunofluorescent analysis to assess cellular localization and relative cell cycle expression using the methods described for the synchrony model above with the addition of 4′,6-diamidino-2-phenylindole (DAPI) staining during secondary antibody incubation in order to judge the qualitative changes in DNA content in the parasite clones.

## Supporting Information

Table S1 (0.11 MB DOC)

Table S2 (0.05 MB DOC)

Figure S1 DNA content changes of thymidine-synchronized populations. RH^TK+^ parasite cultures were blocked with 10 µM thymidine for 4 h and then released. At one hour intervals post thymidine-release (R0-R12), parasites were harvested and pooled for RNA isolation with a small sample removed for DNA content analysis prior to cell lysis. Genomic DNA in ethanol-fixed parasites was stained with SYTOX-Green and changes in DNA content determined by flow cytometry (FL-1; 10,000 events for each sample). As described previously [23], thymidine-arrested RH^TK+^ parasites are blocked at the G1/S phase boundary (R0) and immediately enter S phase upon drug-release (R1). New daughter parasites start emerging by 3 h post drug-release (R3), and the population enters the S phase of the next cell cycle by 7–8 h post-release. The cell cycle progression is indicated with cell cycle phase repeats R1,R2 = R8,R9, R3 = R10, R4 = R11, and R5 = R12 (C = cytokinesis, M = mitosis, G1 = gap 1 phase, S = DNA synthesis).
(1.20 MB TIF)

Figure S2 Cubic B-spline models and the assignment of cyclic and non-cyclic expression patterns. (A) Four genes illustrate the phase patterns of spline models and the protocol used to assign profiles as potentially cyclic or non-cyclic from the real time course data and 1,000 error simulations. The rationale is described in the Methodssection. The 13 time course data points for each gene and the spline curves computed from these points are shown in black. The superimposed colored lines are the sets of 1,000 spline curves from the error simulations: red: a typical ensemble assigned by the protocol as robustly cyclic with Single-wave phase, blue: 3 ensembles assigned as non-cyclic with Linear phase. The phase types can be visually recognized by curve shape as follows: Single-wave: no more than one peak or trough per approximately 8 hour cycle; ‘Double-wave’: typically showing doublet peaks or peaks/troughs determined only by single data points (these are usually cases of over-fitting); ‘Linear’: straight line fit across the entire time series. All these 4 ensembles show more than one phase. In the red set, the majority of the simulated curves and the model from the original data are Single-wave, with a minority of Double-wave (the latter are the more extreme curves with maxima or minima over-fitted to single data points). In the 3 blue ensembles, various proportions of the 1,000 simulated curves show Linear phase as do the models from the original data, which robustly defines these profiles as non-cyclic even though a mixture of all 3 phases may be present as in the top and bottom sets. (**B**) Cubic B-spline models for a sample of 35 genes from a typical contiguous *Toxoplasma* chromosomal region (around BTUB on Chromosome IX). Red lines are the spline models for 17 genes assigned as cyclic by our protocol, black are 14 assigned as non-cyclic (11 of which show low or baseline mRNA signals). Note that some of the red lines show different amplitudes in the two cycles, although the peak/trough recurrence interval is clear in all cases. The blue lines are 4 cases that met the ANOVA criterion and the spline model criterion for Single-wave phase, but were excluded from the 2,833 genes defined as cyclic because they failed the peak/trough recurrence criterion. Inspection of these and many other examples of spline models showed a high level of concordance between the computational assignments and intuitive biological concepts of cyclic, non-cyclic, or developmental patterns.
(0.92 MB TIF)

Figure S3 Gene expression in the G1 phase correlates with expression profiles of cell cycle mutant 88A5. The expression profile of temperature-arrested cell cycle mutant 88A5 is similar to parasites synchronized in the early G1 period (5–6 h post-thymidine release). Many mRNAs that show altered expression, including those with a rapid decline in early G1, have a similar profile in ts mutant 88A5 grown at the non-permissive temperature for 24 h (N = non-permissive temperature 40°C; compare blue colored genes in lanes 6 and N-88A5). The nearly uniform haploid genomic content of mutant 88A5 when growth arrested at 40°C is strikingly similar to the 6 h post-thymidine release (Fig. S1) indicating these parasites share an early G1 phenotype. The characteristic G1 mRNA expression profile was lost in mutant 88A5 grown at the permissive temperature (P =  34°C permissive temperature) and in a genetically rescued clone (88A5-C) grown at either temperature consistent with the asynchronous growth of parasite populations in these cultures.
(3.26 MB TIF)

Figure S4 YFP tagging of AP2 genetic loci. Genomic DNA from each AP2yfp transgenic clone and the parent RHΔ*ku80* was subjected to PCR analysis to confirm YFP tagging of the authentic AP2 gene locus. Primers #1 and #2 detected homologous recombination for each YFP tagging, while primer pair #1 and #3 identified the presence of the wild type genomic locus. T = individual transgenic genomic DNA, P = RHΔ*ku80* genomic DNA, N = no template DNA. PCR fragment sizes obtained for wild type or recombined genetic locus: AP2VIIa-4yfp, primers #1&2 = 3,616 bp, primers #1&3 = 3,302 bp; AP2VI-1yfp #1&2 = 3,664 bp, #1&3 = 3,311 bp; AP2XI-1yfp #1&2 = 4,542 bp, #1&3 = 4,270 bp; AP2XII-9yfp #1&2 = 4,246 bp #1&3 = 3,961 bp; AP2VIIa-1yfp #1&2 = 3,178 bp #1&3 = 2,914 bp.
(7.39 MB TIF)
