# Supplementary material for: Coordinated Progression through Two Subtranscriptomes Underlies the Tachyzoite Cycle of Toxoplasma gondii
Source: PLoS One. 2010 Aug 26;5(8):e12354. doi: 10.1371/journal.pone.0012354 (PMC2928733; doi:10.1371/journal.pone.0012354)
Supplement: Table S1 — (0.11 MB DOC) [file pone.0012354.s001.doc]

| **Cell cycle regulated AP2 factors (24)** | | | | |  | **Other AP2 factors** | | |
| --- | --- | --- | --- | --- | --- | --- | --- | --- |
| **Product**  **Name** | **ToxoDB**  **Version 5 ID** | **Protein**  **Accession #** | **peak**  **phase** | **max**  **time (h)** |  | **Product**  **Name** | **ToxoDB**  **Version 5 ID** | **Protein Accession #** |
| AP2X-9 | TGME49_015150 | XP_002370817.1 | S | 0.7 ±0.10 |  | **Putative bradyzoite factors (11)** | | |
| AP2XII-2 | TGME49_017700 | XP_002371337.1 | S | 1.24 ±0.25 |  | AP2Ib-1 | TGME49_008020 | XP_002369605.1 |
| AP2IX-4 | TGME49_088950 | XP_002368331.1 | S | 1.42 ±0.15 |  | AP2III-3 | TGME49_099150 | XP_002371783.1 |
| AP2X-5 | TGME49_037090 | XP_002369061.1 | S | 1.46 ±0.16 |  | AP2III-4 | TGME49_099020 | XP_002371770.1 |
| AP2VI-1 | TGME49_040460 | XP_002366665.1 | S | 1.7 ±0.10 |  | AP2VIIb-1 | TGME49_062420 | XP_002365344.1 |
| AP2VIIa-8 | TGME49_082210 | XP_002371427.1 | S/M | 2.06 ±0.05 |  | AP2VIII-6 | TGME49_071030 | XP_002365769.1 |
| AP2XI-1 | TGME49_109410 | XP_002368814.1 | S/M | 2.18 ±0.09 |  | AP2IX-1 | TGME49_067460 | XP_002368814.1 |
| AP2III-2 | TGME49_053380 | XP_002369395.1 | S/M | 2.52 ±.25 |  | AP2IX-2 | TGME49_064590 | XP_002368588.1 |
| AP2IV-4 | TGME49_118470 | XP_002369786.1 | S/M | 2.66 ±.25 |  | AP2IX-9 | TGME49_106620 | XP_002369605.1 |
| AP2XII-9 | TGME49_051740 | XP_002367413.1 | S/M | 2.68 ±.21 |  | AP2X-2 | TGME49_025110 | XP_002366184.1 |
| AP2X-11 | TGME49_015570 | XP_002370876.1 | M | 3.24 ±.06 |  | AP2X-10 | TGME49_015340 | XP_002370853.1 |
| AP2VIIa-1 | TGME49_080470 | XP_002371441.1 | C | 3.36 ±0.10 |  | AP2XII-5 | TGME49_047730 | XP_002367140.1 |
| AP2XI-4 | TGME49_115760 | XP_002364754.1 | C | 3.62 ±0.06 |  | **Putative constitutive factors (27)** | | |
| AP2XII-8 | TGME49_050800 | XP_002367362.1 | C/G1 | 4.04 ±0.05 |  | AP2IV-1 | TGME49_120700 | XP_002369926.1 |
| AP2X-7 | TGME49_014840 | XP_002370805.1 | C/G1 | 4.06 ±0.05 |  | AP2IV-2 | TGME49_120680 | XP_002369924.1 |
| AP2VIIa-6 | TGME49_003050 | XP_002367587.1 | C/G1 | 4.26 ±0.06 |  | AP2IV-5 | TGME49_011720 | XP_002371297.1 |
| AP2XII-4 | TGME49_047700 | XP_002367137.1 | C/G1 | 4.28 ±0.19 |  | AP2V-1 | TGME49_020530 | XP_002371577.1 |
| AP2VIII-4 | TGME49_072710 | XP_002365903.1 | G1 | 4.62 ±0.07 |  | AP2V-2 | TGME49_085890 | XP_002369227.1 |
| AP2VIII-7 | TGME49_069010 | XP_002365594.1 | G1 | 4.98 ±0.13 |  | AP2VI-3 | TGME49_044510 | XP_002366940.1 |
| AP2IV-3 | TGME49_118610 | XP_002369800.1 | G1 | 5.2 ±0.07 |  | AP2VIIa-2 | TGME49_080460 | XP_002371440.1 |
| AP2XI-3 | TGME49_110950 | XP_002364339.1 | G1 | 5.34 ±0.04 |  | AP2VIIa-3 | TGME49_005650 | XP_002367796.1 |
| AP2VIIb-2 | TGME49_062000 | XP_002365322.1 | G1/S | 7.64 ±0.22 |  | AP2VIIa-5 | TGME49_003690 | XP_002367651.1 |
| AP2VIII-5 | TGME49_071200 | XP_002365786.1 | G1/S | 7.7 ±0.19 |  | AP2VIIa-7 | TGME49_002490 | XP_002367534.1 |
| AP2VIIa-4 | TGME49_003710 | XP_002367653.1 | G1/S | 7.84 ±0.13 |  | AP2VIIa-9 | TGME49_082220 | XP_002371428.1 |
| **Table S1 AP2 genes encoded in the *Toxoplasma* genome**  All expression assignments were determined by microarray.  1) See Methods section for cell cycle mRNA expression  2) Bradyzoite/constitutive expression was determined in all  three major lineages grown as tachyzoites or induced to  differentiate (≥2 fold) by pH8.2 or Compound 1 induction  (see ref. #42 or ToxoDB for microarray data)  3) Note: bradyzoite AP2III-3 and 4 were determined by  qPCR of in vivo cysts (Gissot personal communication)  4) No expression AP2 factors have a RMA value of ≤80 in  all tachyzoite and bradyzoite microarray samples.  Gene records for all 68 AP2 genes can be accessed at ToxoDB  (http://www.toxodb.org/toxo/). | | | | |  | AP2VIIb-3 | TGME49_055220 | XP_002364851.1 |
|  |  |  |  |  |  | AP2VIII-1 | TGME49_029370 | XP_002367881.1 |
|  |  |  |  |  |  | AP2VIII-2 | TGME49_033120 | XP_002368171.1 |
|  |  |  |  |  |  | AP2VIII-3 | TGME49_073660 | XP_002365967.1 |
|  |  |  |  |  |  | AP2IX-3 | TGME49_064480 | XP_002368587.1 |
|  |  |  |  |  |  | AP2IX-5 | TGME49_089710 | XP_002368396.1 |
|  |  |  |  |  |  | AP2IX-6 | TGME49_090180 | XP_002368433.1 |
|  |  |  |  |  |  | AP2IX-8 | TGME49_106000 | XP_002370372.1 |
|  |  |  |  |  |  | AP2X-1 | TGME49_027900 | XP_002366413.1 |
|  |  |  |  |  |  | AP2X-3 | TGME49_024230 | XP_002366108.1 |
|  |  |  |  |  |  | AP2X-4 | TGME49_024050 | XP_002366091.1 |
|  |  |  |  |  |  | AP2X-8 | TGME49_014960 | XP_002370817.1 |
|  |  |  |  |  |  | AP2XI-2 | TGME49_110900 | XP_002364334.1 |
|  |  |  |  |  |  | AP2XI-5 | TGME49_016220 | XP_002370932.1 |
|  |  |  |  |  |  | AP2XII-1 | TGME49_018960 | XP_002370670.1 |
|  |  |  |  |  |  | AP2XII-6 | TGME49_049190 | XP_002367253.1 |
|  |  |  |  |  |  | **AP2 factors not expressed (6)** | | |
|  |  |  |  |  |  | AP2III-1 | TGME49_052370 | XP_002369345.1 |
|  |  |  |  |  |  | AP2VI-2 | TGME49_040900 | XP_002366709.1 |
|  |  |  |  |  |  | AP2IX-7 | TGME49_090630 | XP_002368457.1 |
|  |  |  |  |  |  | AP2X-6 | TGME49_037440 | XP_002369086.1 |
|  |  |  |  |  |  | AP2XII-3 | TGME49_046660 | XP_002367054.1 |
|  |  |  |  |  |  | AP2XII-7 | TGME49_050070 | XP_002367339.1 |
