# Supplementary material for: Coordinated Progression through Two Subtranscriptomes Underlies the Tachyzoite Cycle of Toxoplasma gondii
Source: PLoS One. 2010 Aug 26;5(8):e12354. doi: 10.1371/journal.pone.0012354 (PMC2928733; doi:10.1371/journal.pone.0012354)
Supplement: Table S2 — (0.05 MB DOC) [file pone.0012354.s002.doc]

| **Table S2** | |
| --- | --- |
| **Gene ID** | **Description** |
| **cyclical chromatin modifiers** | |
| TGME49_093370 | PRMT2 |
| TGME49_107010 | JmjC domain, putative |
| TGME49_091050 | Snf1, putative |
| TGME49_016080 | SET, putative |
| TGME49_094610 | SET, putative |
| TGME49_029460 | SWI2/SNF2-containing protein, putative |
| TGME49_073780 | SWI2/SNF2-containing protein, putative |
| TGME49_118480 | SWI2/SNF2-containing protein, RAD5, putative |
| TGME49_027660 | DNA methyltransferase 2, putative |
| TGME49_052420 | PRMT3 |
| TGME49_094270 | PRMT4/CARM1 H3R17 |
| TGME49_015560 | PRMT5 H3R2 |
| TGME49_019520 | CARM1-like (PRMT6/8) |
| TGME49_081420 | HDAC1 |
| TGME49_002230 | HDAC5 |
| TGME49_040840 | JmjC domain, putative |
| TGME49_042420 | LSD2, putative |
| TGME49_061260 | JMJD6 H3R2 |
| TGME49_083890 | JmjC domain, putative |
| TGME49_046910 | SET, putative |
| TGME49_057770 | SET2 H3K36 |
| TGME49_062750 | SET, putative |
| TGME49_119660 | SET, putative |
| TGME49_088330 | SET, putative |
| TGME49_095610 | SET, putative |
| TGME49_001250 | SET, putative |
| TGME49_011730 | SET8 H4K20 |
| TGME49_018230 | SET, putative |
| TGME49_063420 | Ubiquitin carboxyl-terminal hydrolase, Ubp8, putative |
| TGME49_105480 | Elp3, putative |
| **cyclical C2H2 ZnF factors** | |
| TGME49_ 086710 | zinc finger (C2H2 type) protein |
| TGME49_ 002470 | zinc finger (C2H2 type) domain |
| TGME49_ 059650 | zinc finger (C2H2 type) protein |
| TGME49_ 023570 | zinc finger (C2H2 type) protein |
