# Supplementary figures and images for: Coordinated Progression through Two Subtranscriptomes Underlies the Tachyzoite Cycle of Toxoplasma gondii
Source: PLoS One. 2010 Aug 26;5(8):e12354. doi: 10.1371/journal.pone.0012354 (PMC2928733; doi:10.1371/journal.pone.0012354)

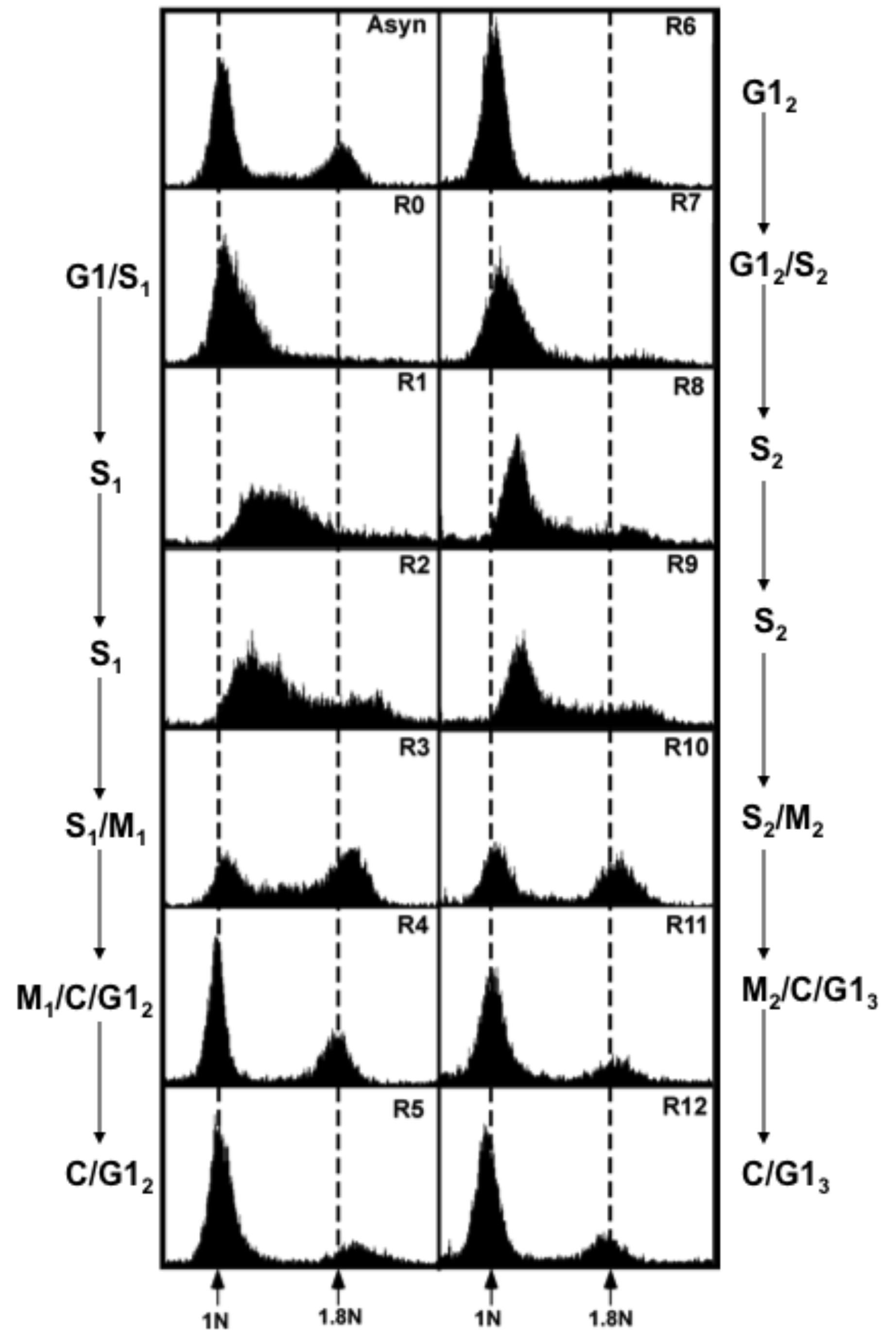

Supplement: Figure S1 — DNA content changes of thymidine-synchronized populations. RHTK+ parasite cultures were blocked with 10 µM thymidine for 4 h and then released. At one hour intervals post thymidine-release (R0-R12), parasites were harvested and pooled for RNA isolation with a small sample removed for DNA content analysis prior to cell lysis. Genomic DNA in ethanol-fixed parasites was stained with SYTOX-Green and changes in DNA content determined by flow cytometry (FL-1; 10,000 events for each sample). As described previously [23], thymidine-arrested RHTK+ parasites are blocked at the G1/S phase boundary (R0) and immediately enter S phase upon drug-release (R1). New daughter parasites start emerging by 3 h post drug-release (R3), and the population enters the S phase of the next cell cycle by 7–8 h post-release. The cell cycle progression is indicated with cell cycle phase repeats R1,R2 = R8,R9, R3 = R10, R4 = R11, and R5 = R12 (C = cytokinesis, M = mitosis, G1 = gap 1 phase, S = DNA synthesis). (1.20 MB TIF) [file pone.0012354.s003.tif]

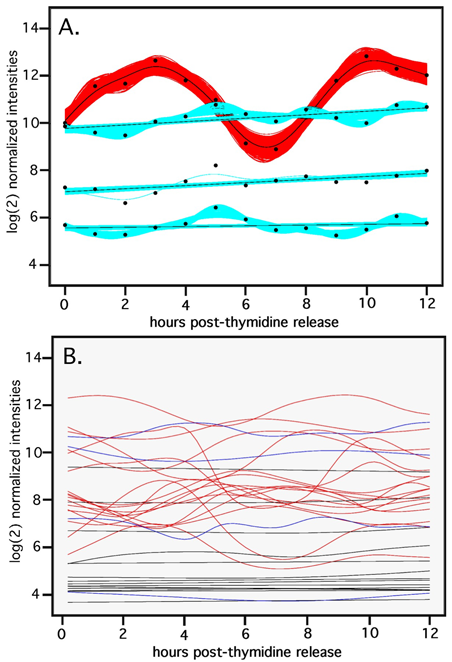

Supplement: Figure S2 — Cubic B-spline models and the assignment of cyclic and non-cyclic expression patterns. (A) Four genes illustrate the phase patterns of spline models and the protocol used to assign profiles as potentially cyclic or non-cyclic from the real time course data and 1,000 error simulations. The rationale is described in the Methodssection. The 13 time course data points for each gene and the spline curves computed from these points are shown in black. The superimposed colored lines are the sets of 1,000 spline curves from the error simulations: red: a typical ensemble assigned by the protocol as robustly cyclic with Single-wave phase, blue: 3 ensembles assigned as non-cyclic with Linear phase. The phase types can be visually recognized by curve shape as follows: Single-wave: no more than one peak or trough per approximately 8 hour cycle; ‘Double-wave’: typically showing doublet peaks or peaks/troughs determined only by single data points (these are usually cases of over-fitting); ‘Linear’: straight line fit across the entire time series. All these 4 ensembles show more than one phase. In the red set, the majority of the simulated curves and the model from the original data are Single-wave, with a minority of Double-wave (the latter are the more extreme curves with maxima or minima over-fitted to single data points). In the 3 blue ensembles, various proportions of the 1,000 simulated curves show Linear phase as do the models from the original data, which robustly defines these profiles as non-cyclic even though a mixture of all 3 phases may be present as in the top and bottom sets. (B) Cubic B-spline models for a sample of 35 genes from a typical contiguous Toxoplasma chromosomal region (around BTUB on Chromosome IX). Red lines are the spline models for 17 genes assigned as cyclic by our protocol, black are 14 assigned as non-cyclic (11 of which show low or baseline mRNA signals). Note that some of the red lines show different amplitudes in the two cycles, although the pea [file pone.0012354.s004.tif]

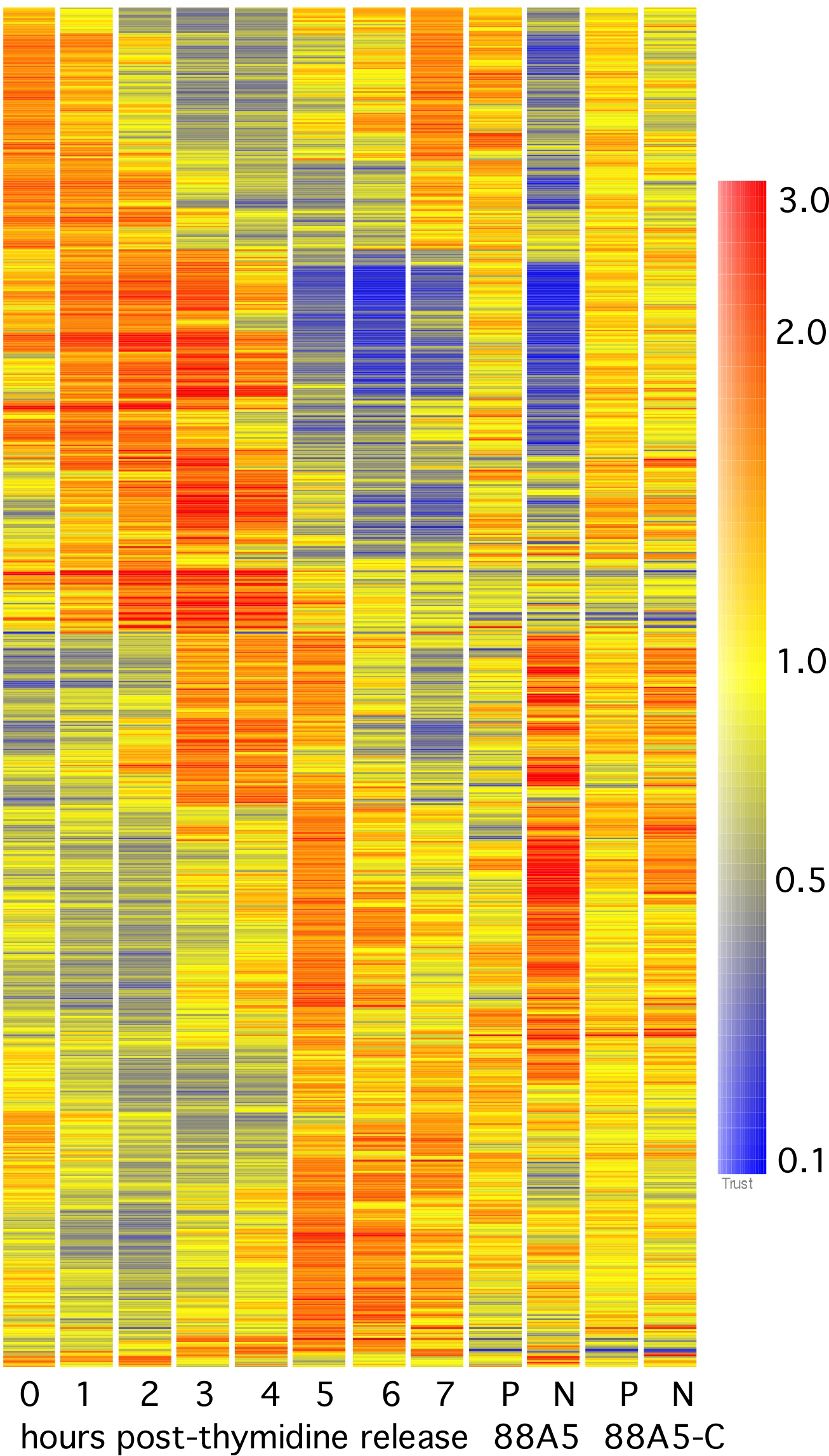

Supplement: Figure S3 — Gene expression in the G1 phase correlates with expression profiles of cell cycle mutant 88A5. The expression profile of temperature-arrested cell cycle mutant 88A5 is similar to parasites synchronized in the early G1 period (5–6 h post-thymidine release). Many mRNAs that show altered expression, including those with a rapid decline in early G1, have a similar profile in ts mutant 88A5 grown at the non-permissive temperature for 24 h (N = non-permissive temperature 40°C; compare blue colored genes in lanes 6 and N-88A5). The nearly uniform haploid genomic content of mutant 88A5 when growth arrested at 40°C is strikingly similar to the 6 h post-thymidine release (Fig. S1) indicating these parasites share an early G1 phenotype. The characteristic G1 mRNA expression profile was lost in mutant 88A5 grown at the permissive temperature (P = 34°C permissive temperature) and in a genetically rescued clone (88A5-C) grown at either temperature consistent with the asynchronous growth of parasite populations in these cultures. (3.26 MB TIF) [file pone.0012354.s005.tif]

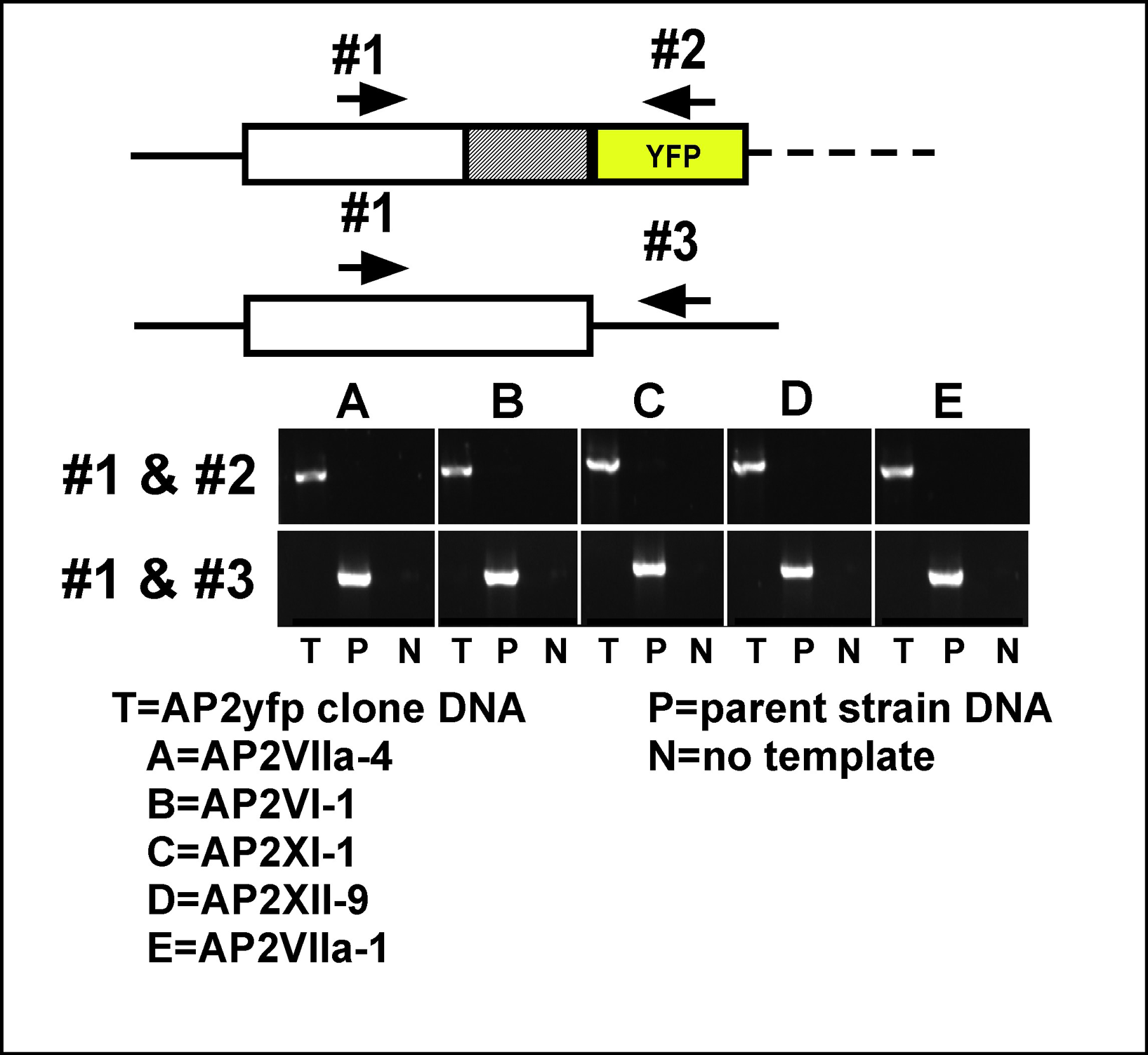

Supplement: Figure S4 — YFP tagging of AP2 genetic loci. Genomic DNA from each AP2yfp transgenic clone and the parent RHΔku80 was subjected to PCR analysis to confirm YFP tagging of the authentic AP2 gene locus. Primers #1 and #2 detected homologous recombination for each YFP tagging, while primer pair #1 and #3 identified the presence of the wild type genomic locus. T = individual transgenic genomic DNA, P = RHΔku80 genomic DNA, N = no template DNA. PCR fragment sizes obtained for wild type or recombined genetic locus: AP2VIIa-4yfp, primers #1&2 = 3,616 bp, primers #1&3 = 3,302 bp; AP2VI-1yfp #1&2 = 3,664 bp, #1&3 = 3,311 bp; AP2XI-1yfp #1&2 = 4,542 bp, #1&3 = 4,270 bp; AP2XII-9yfp #1&2 = 4,246 bp #1&3 = 3,961 bp; AP2VIIa-1yfp #1&2 = 3,178 bp #1&3 = 2,914 bp. (7.39 MB TIF) [file pone.0012354.s006.tif]
